# Structural insight into G-protein chaperone-mediated maturation of a bacterial adenosylcobalamin-dependent mutase

**DOI:** 10.1016/j.jbc.2023.105109

**Published:** 2023-07-28

**Authors:** Francesca A. Vaccaro, Daphne A. Faber, Gisele A. Andree, David A. Born, Gyunghoon Kang, Dallas R. Fonseca, Marco Jost, Catherine L. Drennan

**Affiliations:** 1 Department of Chemistry, Massachusetts Institute of Technology, Cambridge, Massachusetts, USA; 2 Department of Biological Engineering, Massachusetts Institute of Technology, Cambridge, Massachusetts, USA; 3 Graduate Program in Biophysics, Harvard University, Cambridge, Massachusetts, USA; 4 Department of Biology, Massachusetts Institute of Technology, Cambridge, Massachusetts, USA; 5 Amgen Scholar Program, Massachusetts Institute of Technology, Cambridge, Massachusetts, USA; 6 Howard Hughes Medical Institute, Massachusetts Institute of Technology, Cambridge, Massachusetts, USA

**Keywords:** cofactor delivery, metalloenzyme, chaperone, adenosylcobalamin (AdoCbl), GTPase, cryo-electron microscopy, maturation

## Abstract

G-protein metallochaperones are essential for the proper maturation of numerous metalloenzymes. The G-protein chaperone MMAA in humans (MeaB in bacteria) uses GTP hydrolysis to facilitate the delivery of adenosylcobalamin (AdoCbl) to AdoCbl-dependent methylmalonyl-CoA mutase, an essential metabolic enzyme. This G-protein chaperone also facilitates the removal of damaged cobalamin (Cbl) for repair. Although most chaperones are standalone proteins, isobutyryl-CoA mutase fused (IcmF) has a G-protein domain covalently attached to its target mutase. We previously showed that dimeric MeaB undergoes a 180° rotation to reach a state capable of GTP hydrolysis (an active G-protein state), in which so-called switch III residues of one protomer contact the G-nucleotide of the other protomer. However, it was unclear whether other G-protein chaperones also adopted this conformation. Here, we show that the G-protein domain in a fused system forms a similar active conformation, requiring IcmF oligomerization. IcmF oligomerizes both upon Cbl damage and in the presence of the nonhydrolyzable GTP analog, guanosine-5'-[(β,γ)-methyleno]triphosphate, forming supramolecular complexes observable by mass photometry and EM. Cryo-EM structural analysis reveals that the second protomer of the G-protein intermolecular dimer props open the mutase active site using residues of switch III as a wedge, allowing for AdoCbl insertion or damaged Cbl removal. With the series of structural snapshots now available, we now describe here the molecular basis of G-protein–assisted AdoCbl-dependent mutase maturation, explaining how GTP binding prepares a mutase for cofactor delivery and how GTP hydrolysis allows the mutase to capture the cofactor.

With 30 to 50% of the proteome predicted to be metalloproteins, proper maturation of metalloproteins is a vital and nontrivial biological process (1). Metallochaperones are essential for the correct maturation of metalloproteins, ensuring that valuable metallocofactors are delivered efficiently and with minimal toxicity and degradation (2, 3, 4). A prominent class of metallochaperones is composed of the guanine nucleotide–binding proteins (G-proteins) belonging to the SIMIBI (signal recognition particle, MinD, and BioD) class of P-loop NTPases (5). In the absence of their target protein, G-protein metallochaperones generally have low GTP hydrolysis activity. However, target protein binding stimulates GTPase activity by ∼100-fold (6, 7). Some members of this class, such as UreG and HypB, directly bind their metallocofactors and then use GTP hydrolysis to facilitate the maturation of their targets, urease and hydrogenase, respectively (8, 9, 10, 11). Others, like MeaB (MMAA in humans), do not bind directly to their metallocofactor, which is coenzyme B_12_ (5′-deoxyadenosylcobalamin or AdoCbl) in the case of MeaB/MMAA, but still use GTP hydrolysis to deliver AdoCbl to the enzyme target, methylmalonyl-CoA mutase (MCM) (7, 12). In particular, an adenosyltransferase (ATR) adenylates cob(II)alamin using ATP, and MeaB/MMAA facilitates the transfer of AdoCbl from ATR to the cobalamin(Cbl)-binding domain of MCM (13, 14). Typically, G-protein metallochaperones are standalone proteins, with the notable exception of AdoCbl-dependent isobutyryl-CoA mutase fused (IcmF), in which the G-protein metallochaperone exists as a domain of the target enzyme (15). Mutations or deletions in the genes encoding metallochaperones can impair metalloprotein function *in vivo* and lead to disease in humans. For example, in humans, mutations to the genes for MCM, MMAA, or any other chaperones involved in B_12_ trafficking can result in methylmalonic aciduria, an inborn error of metabolism (14, 16).

The AdoCbl cofactor is essential for the chemically challenging carbon skeletal rearrangements that are performed by mutases (17). For MCM, the radical reservoir of the cobalt–carbon bond of AdoCbl catalyzes the 1,2-rearrangement of (*R*)-methylmalonyl-CoA to succinyl-CoA (Fig. 1*A*) (18). IcmF catalyzes the 1,2-rearrangement of isobutyryl-CoA to *n*-butyryl-CoA, as well as pivalyl-CoA and isovaleryl-CoA, using AdoCbl (Fig. 1*A*) (15, 19, 20). The homolytic cleavage of the cobalt–carbon bond of AdoCbl generates cob(II)alamin and a highly reactive 5′-deoxyadenosyl radical species (21). After catalysis, these species must come together to regenerate AdoCbl (Fig. S1); however, if the 5′-deoxyadenosine moiety is lost and/or the cob(II)alamin species is oxidized to hydroxocobalamin (OHCbl), the reformation of AdoCbl is prevented, inactivating the enzyme (20). In addition to these, AdoCbl is also susceptible to inactivation by photolysis of the cobalt–carbon bond (22). To restore activity, the G-protein metallochaperone facilitates the removal of the damaged cofactor and the insertion of a new cofactor from ATR (12, 23, 24). In the active conformation of all known AdoCbl-dependent mutases, AdoCbl is positioned at the interface of a Cbl-binding Rossmann domain and a substrate-binding TIM barrel to afford the generation of substrate-radical species (Figs. 1*B* and S1) (25, 26, 27). In this active state, AdoCbl is sequestered. Thus, AdoCbl delivery to the mutase requires a transient opening and closing of the mutase structure, a conformational change that is believed to be facilitated by MeaB/MMAA (24, 28, 29).

**Figure 1:**
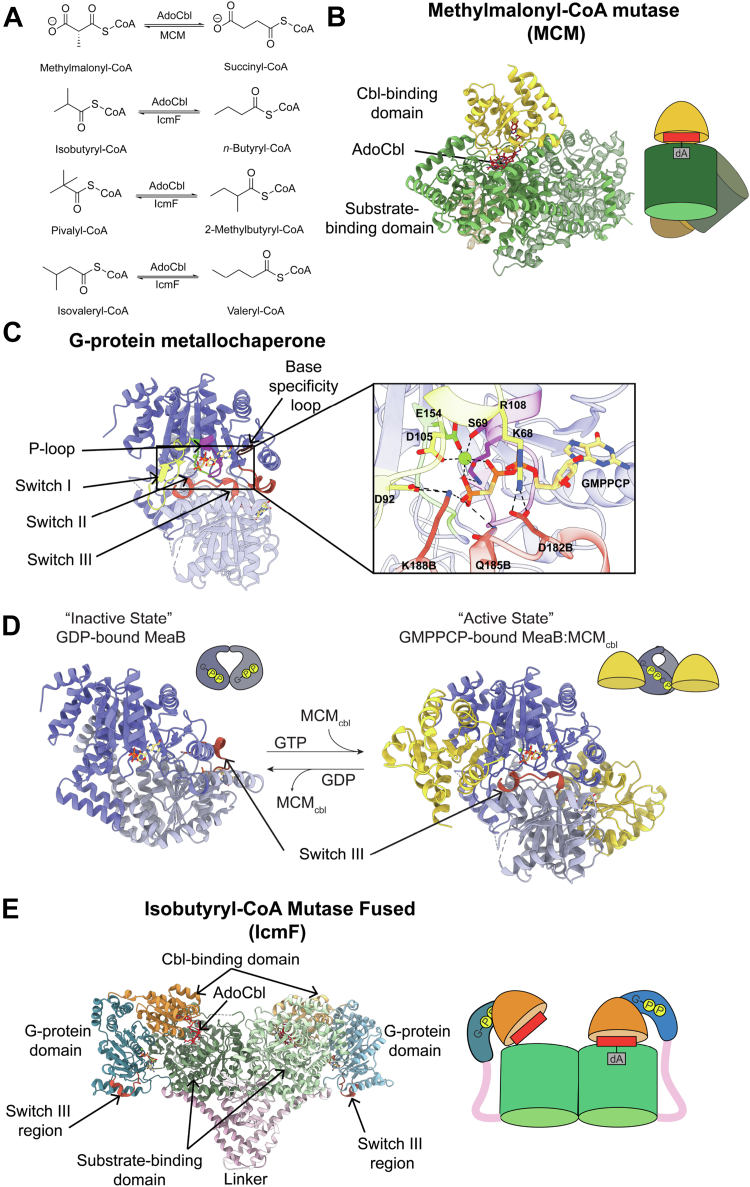
**Adenosylcobalamin-dependent mutase reactions and structures.***A*, AdoCbl-dependent mutase reactions. *B*, methylmalonyl-CoA mutase (PDB 4REQ) (26) consists of a Cbl-binding domain (*yellow ribbons*) and substrate-binding domain (*green ribbons*) with the AdoCbl cofactor (*red sticks*) binding at the interface. The inactive subunit consists of a substrate-binding domain (*light green ribbons*) and Cbl-binding domain (*tan ribbons*) which cannot perform catalysis. Cartoon representation of MCM is colored according to the ribbon drawings. *C*, the G-protein chaperone MeaB (PDB 8DPB) (33) (*purple ribbons*) has conserved P-loop GTPase motifs as labeled and an additional switch III motif (*red orange*) implicated in GTP hydrolysis. Inset: GMPPCP and the Mg^2+^ ion interact with the phosphate-binding loop (*purple sticks*: K68 and S69), switch I region (*yellow sticks*: D92, D105, R108), switch II region (*green sticks*: E154), and the switch III region from the other protomer (*red-orange sticks*: K188, Q185, D182). Residues labeled with “B” are from the other protomer. *D*, MeaB conformational change. Inactive state: PDB 2QM7 (32); Active state: PDB 8DPB (33). Switch III region of the *light purple* MeaB protomer (chain B) rearranges from being solvent-exposed in the “inactive state” to contacting GMPPCP in the “active state”. *E*, *Cupriavidus metallidurans* IcmF (PDB 4XC6) (29) contains two protomers each consisting of a G-protein domain (*blue*) and mutase domain (substrate-binding domain (*green*) and Cbl-binding domain (*orange*)) connected by a linker (*pink*). AdoCbl (*red sticks*) is bound at the active site which is located at the interface of the Cbl-binding domain and substrate-binding domain. The switch III residues are highlighted in *red-orange*. AdoCbl, adenosylcobalamin; Cbl, cobalamin; GMPPCP, guanosine-5'-[(β,γ)-methyleno]triphosphate; IcmF, isobutyryl-CoA mutase fused; MCM, methylmalonyl-CoA mutase.

Consistent with their classifications as P-loop NTPases, MeaB/MMAA and the G-protein domain of IcmF all have a P-loop, a base-specificity loop, and two conserved switch regions for signal transduction: switch I (residues 92–108 in MeaB) and switch II (residues 154–158 in MeaB) (Fig. 1*C*) (6, 30, 31, 32). Unique to MeaB and MMAA is a so-called switch III region (31), which was identified through investigation of residues associated with methylmalonic aciduria (16). *In vitro*, the substitution of MeaB switch III residues Lys188, Gln185, or Asp182 with alanine (Fig. 1*C*) reduces the stimulatory effect in GTP hydrolysis afforded by mutase binding, that is, the GTPase-accelerating protein (GAP) activity, and also leads to an uncoupling of GTP hydrolysis from AdoCbl transfer (31). In the absence of its target mutase, the switch III region of MeaB points toward solution (Fig. 1*D*). However, following the binding of MeaB to the Cbl-binding domain of MCM in the presence of a nonhydrolyzable analog of GTP, guanosine-5'-[(β,γ)-methyleno]triphosphate (GMPPCP), MeaB undergoes a 180° rotation that results in the repositioning of switch III residues directly into the GTP-binding site (Fig. 1*D*) (33). Importantly, it is the switch III residues of the neighboring protomer that contact GTP in this active conformation, indicating the importance of the dimeric structure of MeaB to its function (Fig. 1, *C* and *D*) (33).

The recent structure of GMPPCP-MeaB bound to the Cbl-binding domain of MCM (GMPPCP-MeaB:MCM_Cbl_) led to a proposed molecular mechanism for MeaB function (Fig. 2*A*) (33). In this proposal, the association of GTP-MeaB with the Cbl-binding domain of MCM leads to a conformational change of MeaB from an inactive to active state. The MeaB active state stabilizes an open MCM conformation, allowing MCM to receive AdoCbl from ATR. GTP hydrolysis causes MeaB to undergo a conformational change back to the inactive state. This conformational change of MeaB is proposed to destabilize the open MCM conformation, causing MCM to close and capture AdoCbl inside of the enzyme (Fig. 2*A*).

**Figure 2:**
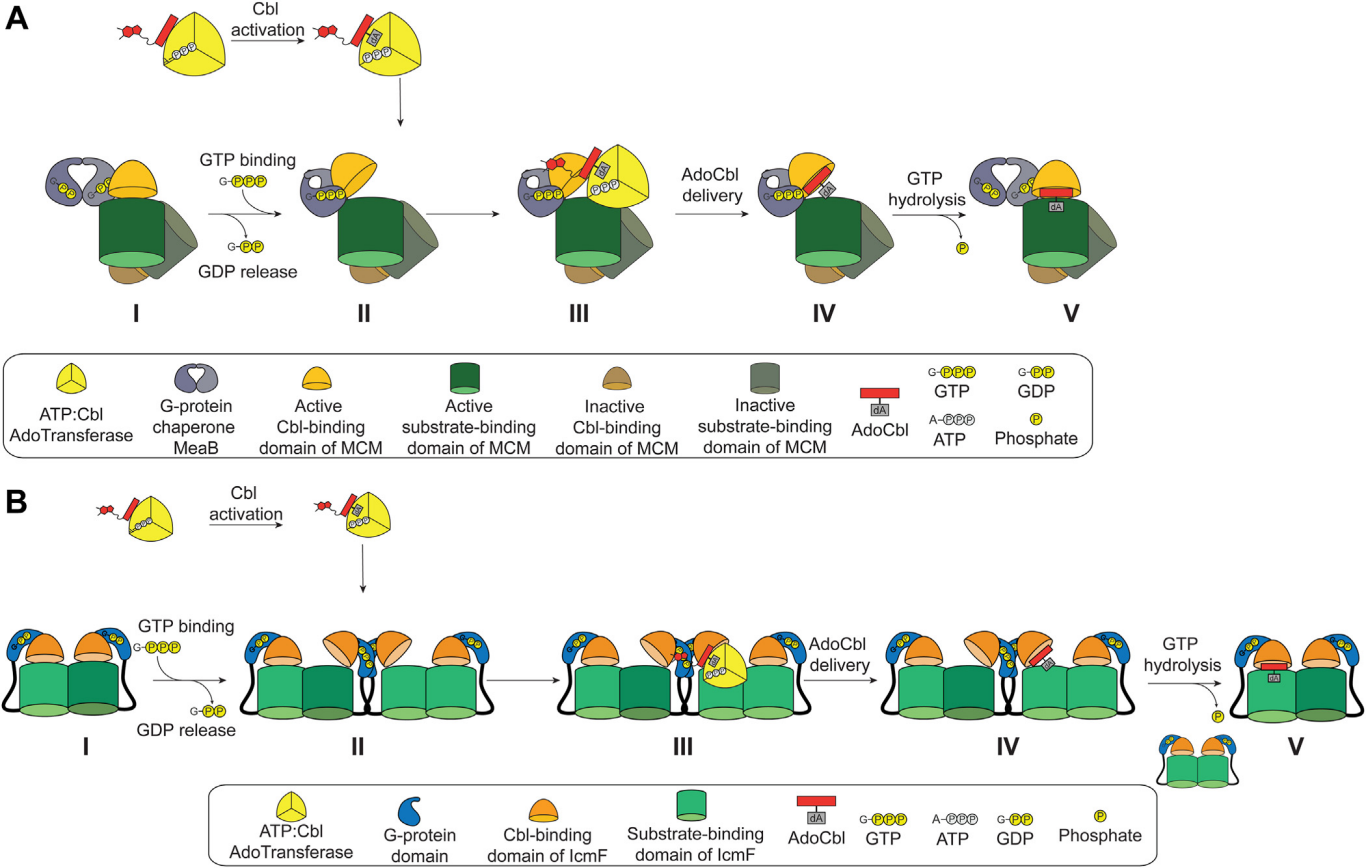
**Proposed steps for loading AdoCbl into the mutase active site.***A*, GTP binding to MeaB leads to the formation of the MeaB “active state”. This conformational change of MeaB opens up one subunit of the MCM heterodimer for AdoCbl delivery from ATR. GTP hydrolysis returns MeaB to the “inactive state,” closing the MCM subunit and capturing AdoCbl. *B*, GTP binding to the G-protein domain of IcmF leads to the formation of a higher order oligomeric state of IcmF, generating a dimer interface analogous to the “active state” of MeaB. Oligomerization of IcmF opens one promoter of the IcmF homodimer for AdoCbl delivery from ATR. GTP hydrolysis breaks apart the higher order oligomer of IcmF, closing the IcmF protomer and trapping the AdoCbl. AdoCbl, adenosylcobalamin; ATR, adenosyltransferase; IcmF, isobutyryl-CoA mutase fused; MCM, methylmalonyl-CoA mutase.

This proposed mechanism (Fig. 2*A*) is supported by crystal structures of GDP-MeaB alone and GMPPCP-MeaB in the presence of MCM_Cbl_ that together reveal the conformational gymnastics involved in the conversion of inactive and active MeaB states (Fig. 1*D*) (32, 33). The connection between GTP hydrolysis and AdoCbl capture, displayed in Figure 2*A* (IV to V), is also supported by biochemical and structural data. Briefly, MCM is unable to capture AdoCbl in the presence of a nonhydrolyzable GTP analog, consistent with the proposal in Figure 2*A* that a GTP hydrolysis–driven MeaB conformational change is needed for MCM to close and thus capture AdoCbl (19, 34). Furthermore, when the active state of MeaB is destabilized by substitutions of switch III residues, such that MeaB can convert to the inactive state independent of GTP hydrolysis, AdoCbl capture is uncoupled from MeaB’s GTPase activity (28, 31). What is missing in terms of experimental support of Figure 2*A* are structures of MCM and MeaB showing that an active MeaB conformation stabilizes an open MCM state in which the Cbl-binding domain of the mutase is positioned away from the substrate-binding domain (I, III, IV in Fig. 2*A*). There is no structure of MeaB bound to an intact MCM; only a structure of MeaB is bound to the Cbl-binding domain of MCM (MCM_Cbl_). Thus, part of the mechanistic proposal in Figure 2*A* is based on structural superpositions of GMPPCP-MeaB:MCM_Cbl_ with the crystal structure of IcmF from *Cupriavidus metallidurans* (29), which contains all relevant structural units: the metallochaperone G-protein domain, the Cbl-binding domain, and the substrate-binding TIM barrel and shares a high level of structural similarity (Figs. 1*E*, S2 and S3). There is, however, the question as to whether IcmF is a good model system for a dimeric MeaB:MCM complex given that the G-protein domains of IcmF are monomeric and located on opposite sides of this large enzyme structure (Fig. 1*E*). IcmF is a dimer, but the dimeric interface is not composed of the G-protein domains (Fig. 1*E*). Thus, if dimerization of the G-protein domains is essential for AdoCbl delivery in IcmF, as it is for MeaB, then IcmF would need to form transient higher-order oligomers (Fig. 2*B*). If oligomers do not form, then IcmF must use a distinct molecular mechanism from MeaB and not what is shown in Figure 2*B*. Also, if IcmF is a good model system for MeaB:MCM, then IcmF should also employ a switch III, a question that has not been investigated.

In this study, we investigate whether IcmF, a fused G-protein system, uses the same molecular mechanism as a standalone G-protein system. By employing site-directed mutagenesis and enzyme assays, we establish that switch III residues are relevant in IcmF from *C. metalliduran*s, and by employing negative stain EM and mass photometry, we show that IcmF oligomerizes as would be expected for a conserved molecular mechanism. With support for a conserved mechanism, we go on to use IcmF to obtain the missing structural snapshot, a mutase bound to a G-protein in a state competent for AdoCbl transfer. With these new data, we describe a consensus molecular mechanism for metallochaperone-assisted AdoCbl-dependent mutase maturation.

## Results

### Substitutions of the switch III regions of IcmF decrease GTPase activity, establishing the relevance of switch III in the IcmF system

**Figure 3:**
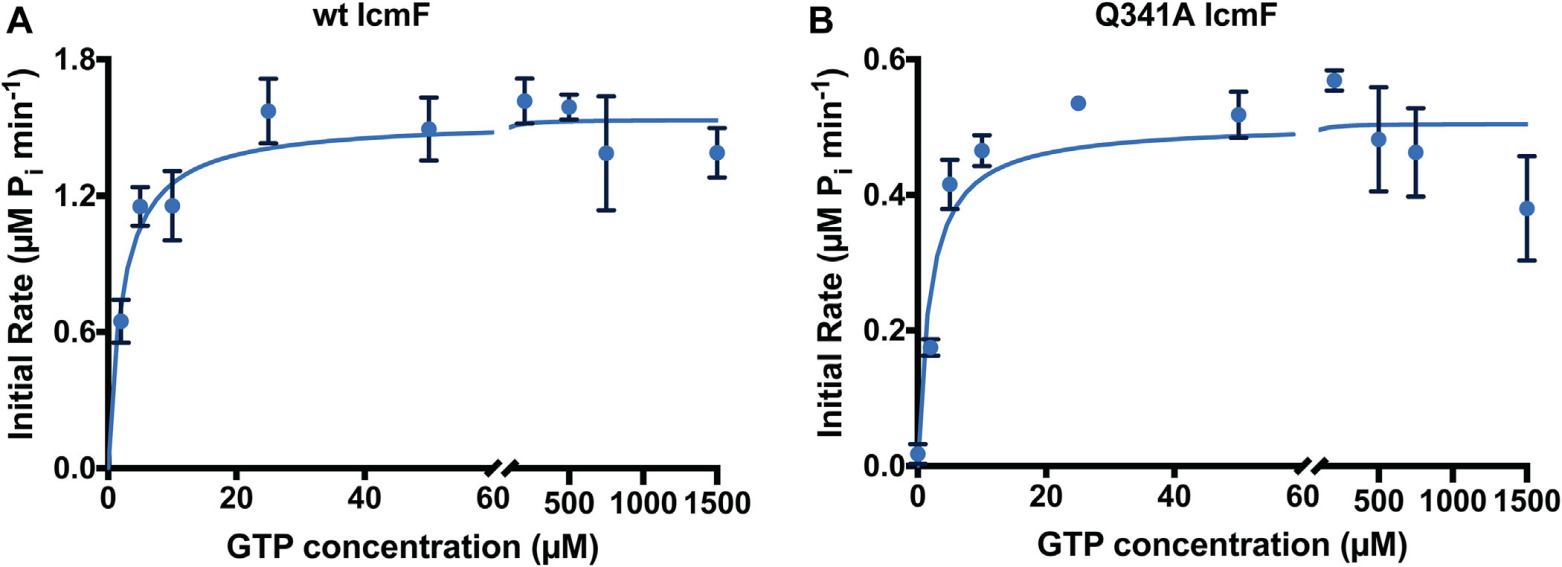
**Michaelis–Menten kinetic analysis of wt IcmF and switch III variant Q341A IcmF.***A*, the GTP hydrolysis activity of wt IcmF. *B*, the GTP hydrolysis activity of the Q341A IcmF variant is lower than that of wt IcmF. Each reaction was performed with 0.5 μM of the enzyme. The data for each curve represent the average ± S.D. of at least three replicates. IcmF, isobutyryl-CoA mutase fused.

To assess if the residues in IcmF that are analogous to the switch III residues of MeaB affect GTPase activity of IcmF as they do in MeaB, we substituted Q341 (Q185 in MeaB) and K344 (K188 in MeaB) with alanine residues and measured GTPase activity (Table 1, Figs. 3 and S3) (31). The GTPase activity of *C. metallidurans* wt IcmF (*k*_*cat*_ = 3.13 ± 0.18 min^−1^) is comparable to previously reported values for IcmF from *C. metallidurans* and *Geobacillus kaustophilus* (Table 1) (15, 20). Substituting the switch III residue Q341 with alanine lowers the catalytic efficiency 2.6-fold mainly through a decrease in *k*_*cat*_ (Table 1). Substituting the switch III residue K344 with alanine lowers the GTPase activity to undetectable levels (Table 1). The observed decrease in GTPase activity of the Q341A and K344A variants is consistent with the observed decrease in the GTPase activity of MeaB switch III variants, validating the importance of the switch III residues in the fused system (31).

**Table 1:** Kinetic parameters for GTPase activity of wt and switch III variants of IcmF and MeaB

Enzyme	*K*_*m*_ GTP μM	*V*_*max*_ μM/min	*k*_*cat*_ min^−1^	Catalytic efficiency *k*_*cat*_*/K*_*m*_ μM min^−1^	Reference
IcmF^a^	2.4 ± 0.9	1.568	3.13 ± 0.18	1.3	This study
	40 ± 8	N.R.^b^	18 ± 1.3	0.45	(20)
*Geobacillus kaustophilus* IcmF	51 ± 3	N.R.	10 ± 1	0.19	(20)
MeaB	N.R.	N.R.	0.039 ± 0.003	N. R.	(31)
MeaB + MCM	N.R.	N.R.	4.20 ± 0.21	N.R.	(31)
Q341A IcmF	2.2 ± 0.8	0.533	1.07 ± 0.07	0.49	This study
Q185A MeaB + MCM	N.R.	N.R.	0.17 ± 0.03	N.R.	(31)
K344A IcmF	N.D.^c^	N.D.	N.D.	N.D.	This study
K188A MeaB + MCM	N.R.	N.R.	0.14 ± 0.02	N.R.	(31)

a Unless noted, all IcmF GTPase activity data is from *Cupriavidus metallidurans*.

b N.R.: Not reported.

c N.D.: Not detected.

### IcmF forms higher order oligomers in the presence of GTP and nonhydrolyzable GTP analogs

**Figure 4:**
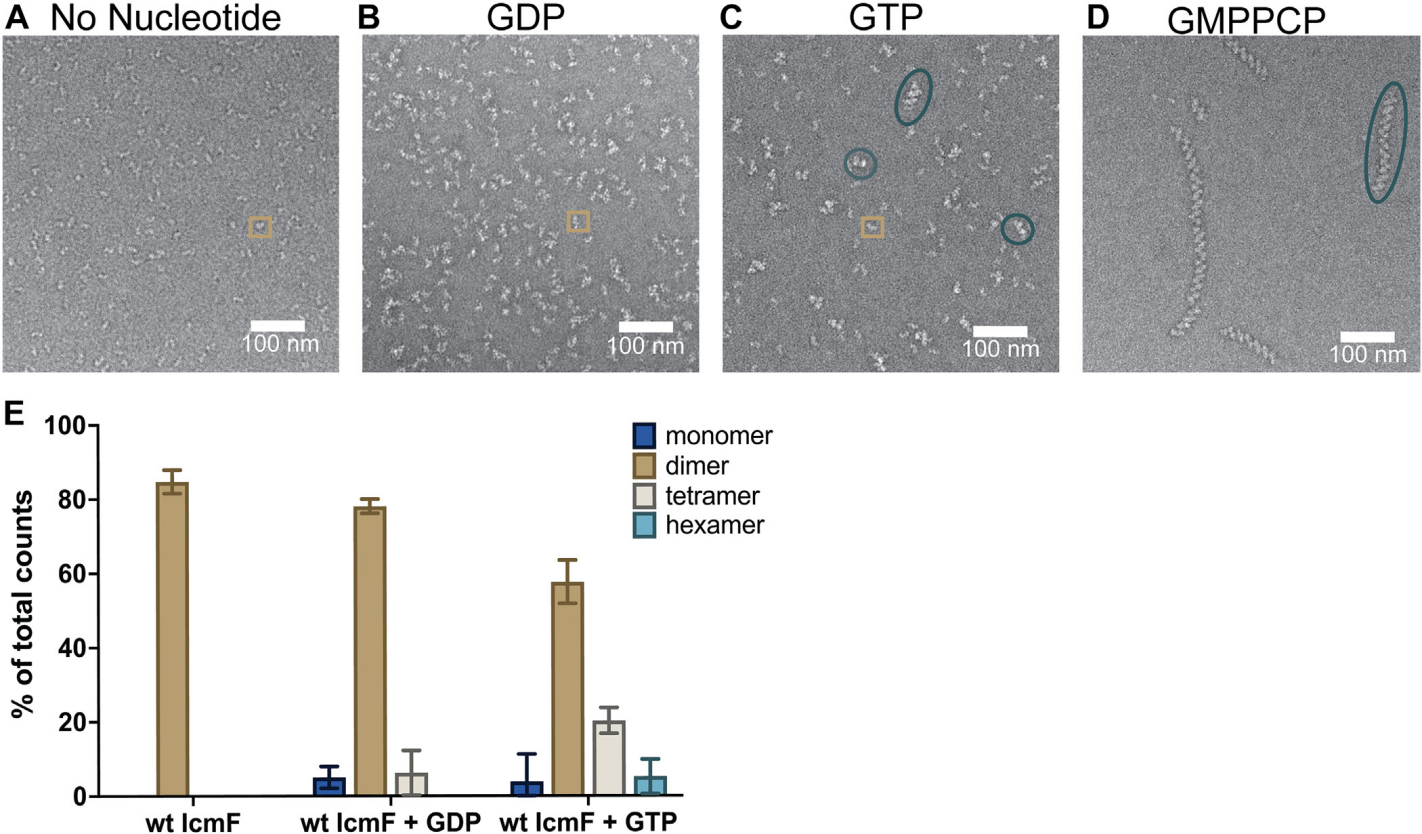
**Negative stain EM and mass photometry analysis of the oligomeric state of wt IcmF in the absence and presence of G-nucleotides.***A*, negative stain EM image of 20 ng/μl wt IcmF without any nucleotide. Representative IcmF dimer is boxed in *tan*. *B*, 20 ng/μl wt IcmF in the presence of 500 μM of GDP. Representative IcmF dimer is boxed in *tan*. *C*, 20 ng/μl wt IcmF in the presence of 500 μM GTP. Representative IcmF dimers are boxed in *tan*, and supramolecular complexes are *circled* in *teal*. *D*, 20 ng/μl wt IcmF in the presence of 500 μM GMPPCP. A representative supramolecular complex is *circled* in *teal*. *E*, mass photometry analysis of wt IcmF without any nucleotide or in the presence of 500 μM GDP or 500 μM GTP indicate the presence of a greater proportion of higher order oligomers in the presence of GTP. The data for each condition represents the mean ± S.D of at least four replicates. The dimensions and heterogeneity in length of the GMPPCP-generated IcmF supramolecular complexes precluded accurate quantification by mass photometry. GMPPCP, guanosine-5'-[(β,γ)-methyleno]triphosphate; IcmF, isobutyryl-CoA mutase fused.

To understand if the fused system also utilizes a G-protein dimer arrangement similar to MeaB and other members of the SIMIBI G-proteins (5), we analyzed the oligomeric state of *C. metallidurans* wt IcmF in solution in the presence and absence of various G-nucleotides. If the fused system’s active state is comparable to the nonfused system, IcmF should form a higher order oligomeric state in the presence of GTP or a nonhydrolyzable analog due to the association of one G-protein domain of an IcmF molecule with the G-protein domain of another. GTP hydrolysis should break apart these higher order oligomers, returning IcmF to the dimeric state that was visualized in the crystal structures (Fig. 1*E*) (29, 35). Thus, we would expect to see more higher-order oligomers with GMPPCP, a nonhydrolyzable analog of GTP. Additionally, we would expect to see only dimers under inactive GTPase conditions, that is, with no nucleotide or with GDP. Importantly, our negative stain EM and mass photometry data are consistent with these predictions (Fig. 4). Negative stain EM analysis indicates that with no nucleotide or in the presence of 500 μM GDP, IcmF does not form any observable higher-order oligomers and remains dimeric (Fig. 4, *A* and *B*). In the presence of 500 μM GTP, the percentage of wt IcmF in a dimeric state decreases and higher order oligomers are observed (Fig. 4*C*). With 500 μM of the nonhydrolyzable GTP analog, GMPPCP, long chains of IcmF protomers are observed, with all visible IcmF protomers comprising these filamentous-like chains (Fig. 4*D*). These supramolecular structures are consistent with IcmF dimers interacting with other IcmF dimers through the surface-exposed G-protein domains on either end of the long IcmF molecule (see Fig. 1*E*).

Using mass photometry to evaluate the oligomers, we also find that in the presence of 500 μM GDP, the primary state of IcmF is dimeric (80%), consistent with the negative stain EM (Fig. 4*E*) and the crystal structure (Fig. 1*E*) (29). In the presence of 500 μM GTP, the percentage of IcmF in a dimeric state is decreased from 80% to 60% and higher order oligomers are observed (22% of particles are tetramers and 7.5% of particles are hexamers). The dimensions and heterogeneity in length of the GMPPCP-generated IcmF supramolecular complexes precluded accurate quantification by mass photometry.

### The identity of the Cbl cofactor influences IcmF’s oligomeric state

According to the mechanism shown in Figure 2*B*, we expect that the G-protein domain of IcmF only dimerizes when IcmF needs to open for removal of a damaged cofactor or installation of a new one. Thus, we investigated whether a correlation exists between the identity of the Cbl cofactor (active AdoCbl or inactive cob(II)alamin or OHCbl) and the oligomeric state of IcmF. We employed negative stain EM exclusively for our studies with Cbl, as the Cbl visual spectra interfered with the light scattering method utilized in mass photometry, preventing the quantitative analysis of oligomeric state by mass photometry that was possible for GDP and GTP. Negative stain EM grids were prepared both under red light (“dark”) and under white light (“light”). Samples prepared under red light should have intact AdoCbl, and experiments conducted in the light should have inactivated OHCbl cofactor due to photolysis of AdoCbl and oxidation of the resulting cob(II)alamin to OHCbl. We also used OHCbl in these studies, which represent the fully inactivated and oxidized AdoCbl degradation product.

**Figure 5:**
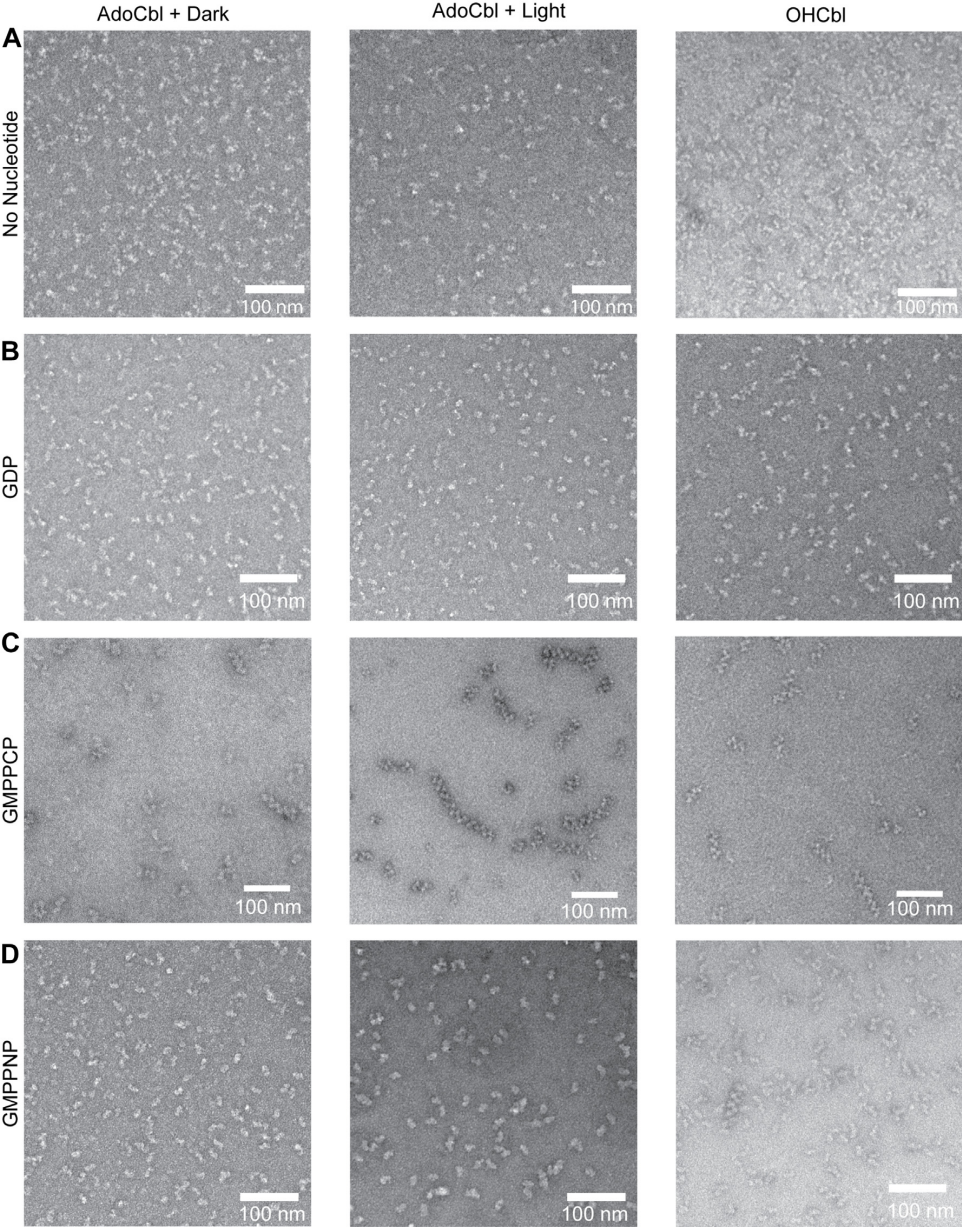
**Negative stain EM analysis of the oligomeric state of IcmF when bound to various states of the cobalamin cofactor in the presence and absence of G-nucleotides.***A*, representative negative stain EM images of 20 ng/μl wt IcmF in the presence of AdoCbl incubated in the dark (*left*), AdoCbl exposed to light (*middle*), and OHCbl (*right*). None of the conditions form supramolecular complexes. *B*, representative negative stain EM images of 20 ng/μl wt IcmF in the presence of AdoCbl and GDP incubated in the dark (*left*), AdoCbl and GDP exposed to light (*middle*), and OHCbl and GDP (*right*). None of the conditions form supramolecular complexes. *C*, representative negative stain EM images of 20 ng/μl wt IcmF in the presence of AdoCbl and GMPPCP incubated in the dark (*left*), AdoCbl and GMPPCP exposed to light (*middle*), and OHCbl and GMPPCP (*right*). All the states contain supramolecular complexes; however, the states with inactivated cofactor (AdoCbl exposed to light or OHCbl) have more supramolecular complexes. The concentration of all cofactors and nucleotides added was 500 μM. *D*, representative negative stain EM images of 20 ng/μl wt IcmF in the presence of AdoCbl and GMPPNP incubated in the dark (*left*), AdoCbl and GMPPNP exposed to light (*middle*), and OHCbl and GMPPNP (*right*). Only the states that have inactivated cofactor (AdoCbl exposed to light or OHCbl) and a nonhydrolyzable analog contain supramolecular complexes. The concentration of all cofactors and nucleotides added was 500 μM. Each negative stain condition was repeated three times. AdoCbl, adenosylcobalamin; GMPPCP, guanosine-5'-[(β,γ)-methyleno]triphosphate; GMPPNP, guanosine-5'-[(β,γ)-imido]triphosphate; IcmF, isobutyryl-CoA mutase fused.

We find that with no nucleotide or with GDP that the identity of the Cbl present (AdoCbl in dark or light or OHCbl) does not alter *C. metalliduran*s IcmF oligomerization by any appreciable extent. IcmF appears to be dimeric in all cases (Fig. 5, *A* and *B*). When GMPPCP is used, supramolecular complexes are observed in all cases. However, more supramolecular complexes are apparent for samples prepared in the light, when more AdoCbl has been photolyzed, and in the presence of OHCbl, than for AdoCbl samples prepared in the dark (Fig. 5*C*). We have previously noted that GMPPCP is more effective at inducing complex formation between MeaB and MCM_Cbl_ in comparison to another nonhydrolyzable GTP analog, guanosine-5'-[(β,γ)-imido]triphosphate (GMPPNP) (33). Thus, we wondered if usage of the weaker oligomerization-agonist GMPPNP would allow us to observe smaller effects on oligomerization that are due to the nature of the Cbl cofactor. We find qualitatively that there are very few, if any, IcmF supramolecular complexes in the presence of GMPPNP when AdoCbl is intact (dark sample) (Fig. 5*D*). Photolysis of AdoCbl leads to more supramolecular complex formation but not as many as in the GMPPNP + OHCbl sample (Fig. 5*D*). Together, these data indicate that IcmF oligomerization depends more strongly on the identity of the G-nucleotide than the identity of the Cbl cofactor, but IcmF with a damaged Cbl is more prone to oligomerization than IcmF with an intact AdoCbl. Thus, both the G-nucleotide identity and the Cbl identity shift the oligomeric state equilibrium, but to different degrees.

### Cryo-EM data reveal a G-protein dimer and an open conformation of the mutase

**Figure 6:**
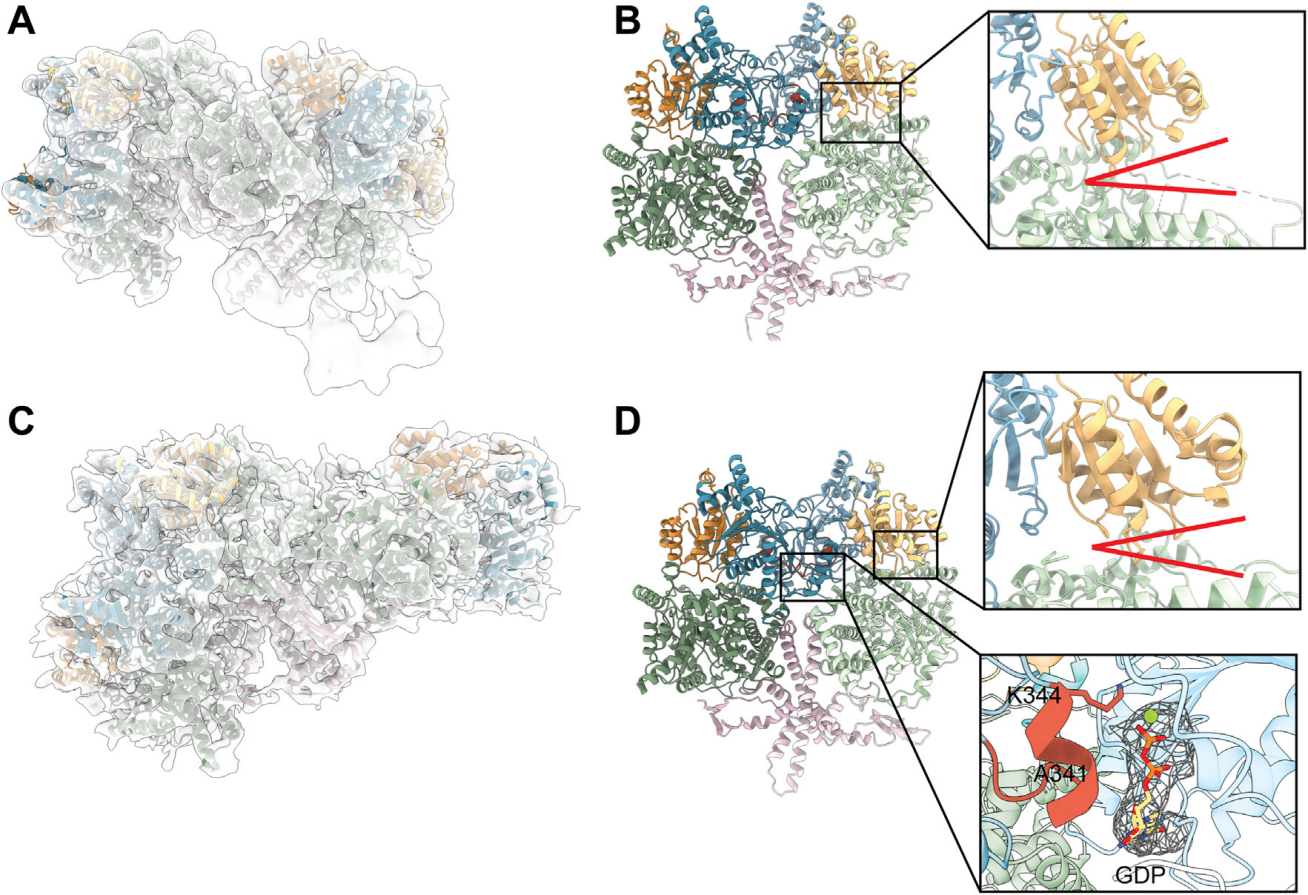
**3D cryogenic EM helical reconstruction of the supramolecular complexes of IcmF indicates the formation of a G-protein interdimer interface in solution.***A*, 6.7-Å resolution single particle reconstruction of the supramolecular complexes of wt IcmF in the presence of 500 μM GMPPCP and 500 μM butyryl-CoA (wt IcmF + GMPPCP) with four IcmF protomers modeled into the EM map. The unmodeled extra density represents additional density from the continuous supramolecular complexes that cannot accommodate a full IcmF protomer. *B*, two of the four protomers from wt IcmF + GMPPCP structure are shown. Each protomer is from a different IcmF homodimer (the homodimer is not shown for simplicity). The majority of the intermolecular contacts are made by the G-protein domains. *Coloring*: G-protein domain in *teal*, the substrate-binding domain in *green*, and the linker region in *pink*. Inset: The mutase active site is open indicated by the *red lines*. *C*, 4.6-Å resolution single particle reconstruction of the complexes of Q341A IcmF in the presence of 500 μM GTP (Q341A IcmF + GTP) with three IcmF protomers modeled into the EM map. *D*, two of the three protomers from Q341A IcmF + GTP structure are shown. Each protomer is from a different IcmF homodimer (the homodimer is not shown for simplicity). As in *B*, the majority of the intermolecular contacts are made by the G-protein domains. Colored as described in *B*. *Top inset*: The mutase active site is open indicated by the *red lines*. *Bottom inset*: Switch III residues (*red*) from one protomer contact the GDP bound to the second protomer across the G-protein domain: G-protein domain interface. GDP and Mg^2+^ ion are shown against the EM map. GMPPCP, guanosine-5'-[(β,γ)-methyleno]triphosphate; IcmF, isobutyryl-CoA mutase fused.

To further investigate the structures of IcmF’s supramolecular complexes, we prepared cryogenic EM grids and collected datasets of *C. metalliduran*s wt IcmF with GMPPCP and butyryl-CoA (wt IcmF + GMPPCP) and Q341A IcmF with GTP (Q341A IcmF + GTP). For the wt IcmF + GMPPCP dataset, the supramolecular complexes were first manually picked on the micrographs, extracted using helical parameters, and processed as a single particle dataset. Signal subtraction was utilized to remove the extra density that was due to the extraction of single particles from a continuous helical particle (Fig. S4) (36). Local B factor postprocessing was performed to yield a 6.7-Å resolution reconstruction (Fig. 6*A*, Table S1) (37). For the Q341A IcmF + GTP dataset, we performed 3D single particle reconstructions, using repeated classifications for both 2D and 3D steps to separate out heterogeneity (Fig. S5). Local B factor postprocessing was also performed to yield a 4.6-Å resolution reconstruction (Fig. 6*C*, Table S1). For each reconstruction, the previously solved crystal structure of *C. metalliduran*s IcmF with GDP and AdoCbl bound was used as the initial docking model (29).

In both reconstructions, one IcmF dimer contacts the other IcmF dimer through intermolecular interactions made by the G-protein domains (cyan in Fig. 6, *B* and *D*). The G-protein domain interface adopts the same conformation as observed for MeaB in the GMPPCP-MeaB:MCM_Cbl_ complex structure. Again, we observe switch III residues positioned at the interface adjacent to the GTP-binding site (Figs. S6*A* and S7). For the Q341A IcmF + GTP reconstruction, there is clear density for the guanosine and at least two phosphates of GTP in all the nucleotide-binding sites (Fig. 6*D*). However, due to the lower resolution of the wt IcmF + GMPPCP reconstruction, there is no clear density for GMPPCP in the nucleotide-binding site. For both reconstructions, we observed an open conformation of the mutase active site (Fig. 6, *B* and *D*).

**Figure 7:**
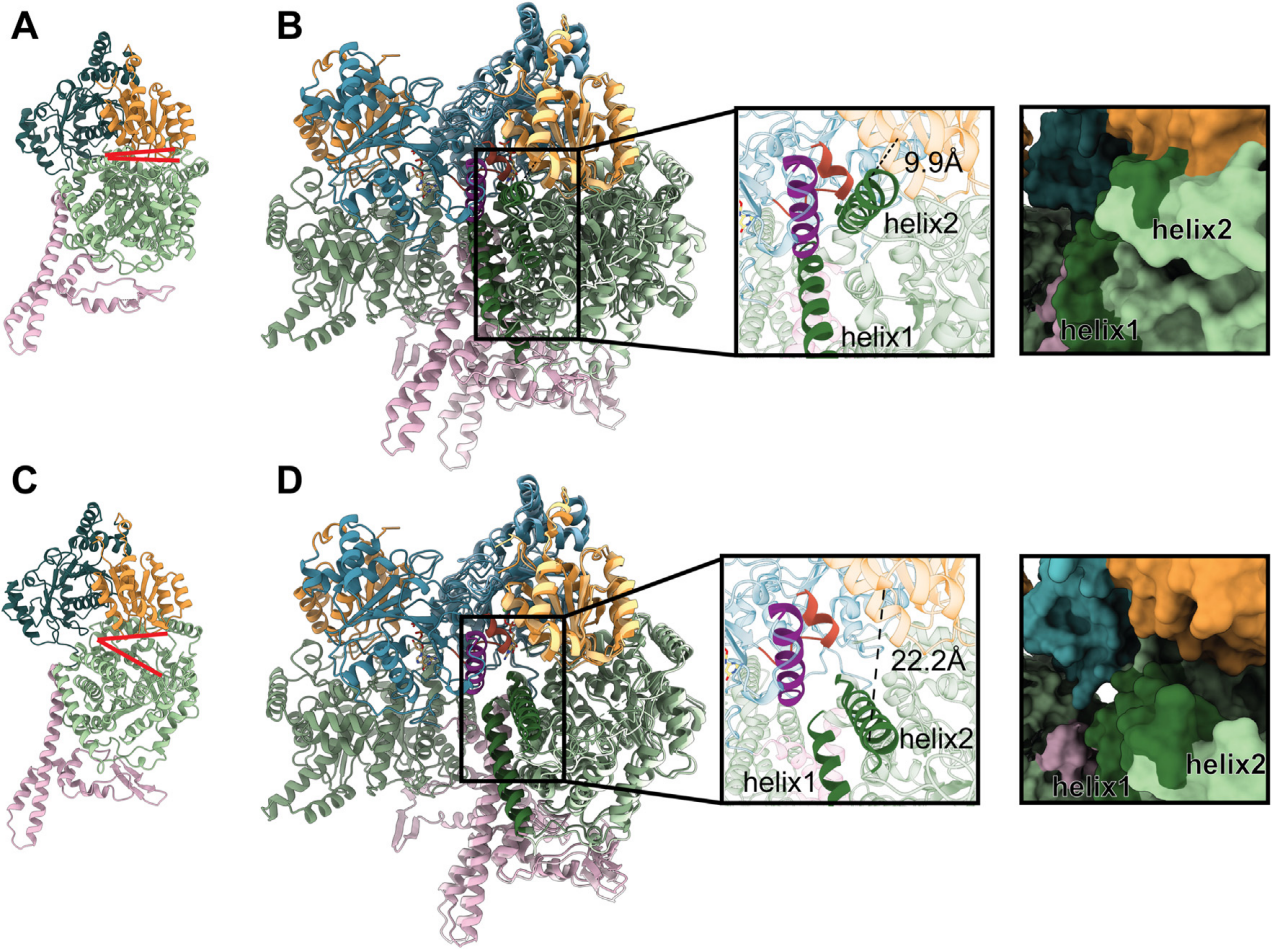
**The G-protein domain interface wedges open the active site of the mutase domain of IcmF.***A*, ribbon drawing of the closed conformation of IcmF (PDB 4XC6) (29) with the substrate-binding domain (*green*) and Cbl-binding domain (*orange*), G-protein domain (*blue*) and linker (*pink*). *Red lines* indicate there is no gap between the domains. *B*, overlay of the closed conformation of IcmF (PDB 4XC6) (29) with the Q341A IcmF + GTP structure. *Left inset*: overlay reveals a clash between the helices of the substrate-binding domain of IcmF (*dark green*, helix 1 residues: 942–965; helix 2 residues: 974–995) with the switch III region (*red*-*orange*, residues 333–344) or helix (*purple*, residues 363–380) from the neighboring IcmF protomer. The distance between helix 2 and the Cbl-binding domain is 9.9 Å. *Right inset*: the same orientation as on *left* as surface representation but without the clashing G-protomer. *C*, ribbon drawing of the open conformation of IcmF (PDB 4XC6) (29) colored as in *A*. *Red lines* indicate there is a gap between the domains. *D*, overlay of the open conformation of IcmF (PDB 4XC6) (29) with the Q341A IcmF + GTP structure. *Left inset*: overlay shows no clashing between the helices of the substrate-binding domain of IcmF (*dark green*, helix 1 residues: 942–965; helix 2 residues: 974–995) with the switch III region (*red-orange*, residues 333–344) or helix (*purple*, residues 363–380) from the neighboring IcmF protomer. The distance between helix 2 and the Cbl-binding domain is 22.2 Å. *Right inset*: the same orientation as on *left* as surface representation but without the wedging G-protomer. Cbl, cobalamin; IcmF, isobutyryl-CoA mutase fused.

Overlaying previously determined closed (Fig. 7*A*) and open (Fig. 7*C*) conformations of IcmF onto the structure of Q341A IcmF + GTP reveals that the G-protein interdimer interface clashes with the closed conformation (Fig. 7*B*). Specifically, the switch III region (residues 333–344) and a helix (purple in Fig. 7*B*, residues 363–380) of one protomer clash with two helices of the substrate-binding domain of the other protomer (helix 1, residues 942–965, and helix 2, residues 974–995 in Fig. 7*B*); these clashes are not observed when the mutase is in an open conformation (Fig. 7*D*). Thus, in addition to switch III residues residing at the interface created by the G-protein domains, they also participate in propping open the mutase (Fig. 7, *B* and *D*). These structures are consistent with the biochemical solution state data, showing that supramolecular complexes form under conditions that require loading or unloading of Cbl. Overall, the cryo-EM reconstructions have trapped the conformation of G-protein domain that wedges open the active site of the mutase domain for cofactor to be loaded and unloaded.

## Discussion

Metallochaperones ensure that valuable, and often highly reactive, metallocofactors are delivered efficiently to their target enzymes. The molecular basis for successful delivery is often enigmatic, especially for metallochaperones like MeaB that do not bind the metallocofactor directly. Because the complexes formed by the metallochaperone and target enzyme are transient and can sample more than one conformational state, understanding the molecular basis of metalloenzyme maturation and/or cofactor repair involves the difficult task of trapping transient protein:protein complexes in multiple conformational states. In this study, we use cryo-EM to capture a long-awaited structure of the active conformation of an AdoCbl metallochaperone in complex with a target mutase and use mutagenesis and mass photometry to understand the assembly/disassembly of active cofactor-transfer complexes. Our data support the existence of a conserved molecular mechanism for AdoCbl transfer between the fused IcmF system and the standalone MeaB:MCM system and provide the snapshots needed to understand the molecular basis of metallochaperone-assisted AdoCbl transfer.

Previous studies showed that the bacterial AdoCbl metallochaperone MeaB is always dimeric and uses a dramatic 180° conformational change to switch between active (GTP-bound) and inactive (GDP-bound) states (Fig. 1*D*) (32, 33). In contrast, we previously showed that the fused metallochaperone:mutase system IcmF has a chaperone G-protein domain that is monomeric in the inactive state (Fig. 1*E*) (29), and we show here that IcmF uses oligomerization to switch between its inactive (GDP-bound) states and the active (GTP-bound) state. Evidence for the coupling of GTP binding to an IcmF oligomeric state change comes from negative stain EM, cryo-EM, and mass photometry (Figs. 4 and 6). Importantly, regardless of whether the active conformation of the G-protein domain is formed through oligomerization (IcmF) or through a conformational change of an obligate dimer (MeaB/MMAA), the interface between protomers is the same (Fig. S7).

The G-protein:G-protein interface is formed in both systems by switch III residues of one protomer and the G-nucleotide and switch I region of the other protomer (Figs. 1*C*, inset, and 6*D*, inset) (33). This G-protein:G-protein interface is small, which allows for modest changes, such as loss of a phosphate group due to GTP hydrolysis, to shift the conformational (or oligomeric state) equilibrium between active and inactive G-protein states. Insight into the molecular basis for this conformational/oligomeric state shift comes from structural comparisons of GMPPCP-bound MeaB:MCM_Cbl_ (33) and GDP-bound MeaB (32), which indicate that GTP hydrolysis would bring about the loss of the contacts made by chain-B switch III residues K188 and Q185 (MeaB numbering) to the chain-A terminal phosphate of GMPPCP and loss of the interchain salt bridge made by switch III residue D182 (chain-B) and switch I residue R108 (chain-A, MeaB numbering) (Fig. 1*C* inset). In terms of the salt bridge, structural comparisons suggest that the repositioning of intradomain MeaB residues D92, E154, and R108 (Fig. 1*C*, inset) as a result of GTP hydrolysis and Mg^2+^ loss in turn repositions R108, breaking the interchain salt bridge (see Fig. S8). Collectively, the loss of the terminal phosphate of GTP results in a loss of all contacts that stabilize the active state of MeaB. Although the resolution of the cryo-EM IcmF structures is too low for a detailed analysis of protein:G-nucleotide interactions, we do find that the switch III residues including K344 (K188 in MeaB) and Q341 (Q185 in MeaB) of one IcmF protomer are in direct contact with the G-nucleotide bound to another IcmF protomer. This arrangement in IcmF is just what one would expect for a conserved mechanism in which the binding/orientation of one G-protein protomer is based on the G-nucleotide–bound state of another G-protein protomer.

Despite the modest resolution of the cryo-EM structures determined in this study, establishing the positioning of the individual domains of IcmF was straightforward (Fig. 6). The resulting model shows that the active G-protein:G-protein interface stabilizes an open conformation of the mutase that is ready for AdoCbl delivery. It was previously proposed through structural superimpositions that the active G-protein conformation would stabilize the Cbl-binding domain away from the substrate-binding domain for cofactor loading in MeaB:MCM (Fig. 2*A*) and similarly in IcmF (Fig. 2*B*) (33) and here we see that this is in fact the case (Fig. 6). The second protomer of the G-domain dimer is wedged between the Cbl-binding domain and the substrate-binding domain, holding the mutase open (Fig. 7*D*, left inset). The closed conformation of the mutase appears incapable of interacting with the active G-protein state based on the structural superimpositions of IcmF structures (Fig. 7*B*, left inset). We previously predicted that the formation of the active state of MCM-bound MeaB would lead to a clash between a helix of MeaB (equivalent to the IcmF helix shown in purple in Fig. 7*B*, left inset) and a helix of the substrate-binding domain (helix 1 in Fig. 7*B*, left inset) if MCM remained in a closed state (33). This prediction is consistent with our current structures. Additionally, structural superimpositions suggest that the switch III region (red in Fig. 7*B*, left inset) also would make unfavorably close interactions with a substrate-binding domain helix (helix 2 in Fig. 7*B*, left inset). This observation provides an additional role for switch III, acting as a molecular wedge that is sensitive to the identity of the G-nucleotide state. The wedge is secured with GTP bound, allowing AdoCbl to be delivered to an open structure, and the wedge is loosened by GTP hydrolysis, allowing the Cbl-binding domain to close, trapping a delivered AdoCbl (Fig. 2*B*).

**Figure 8:**
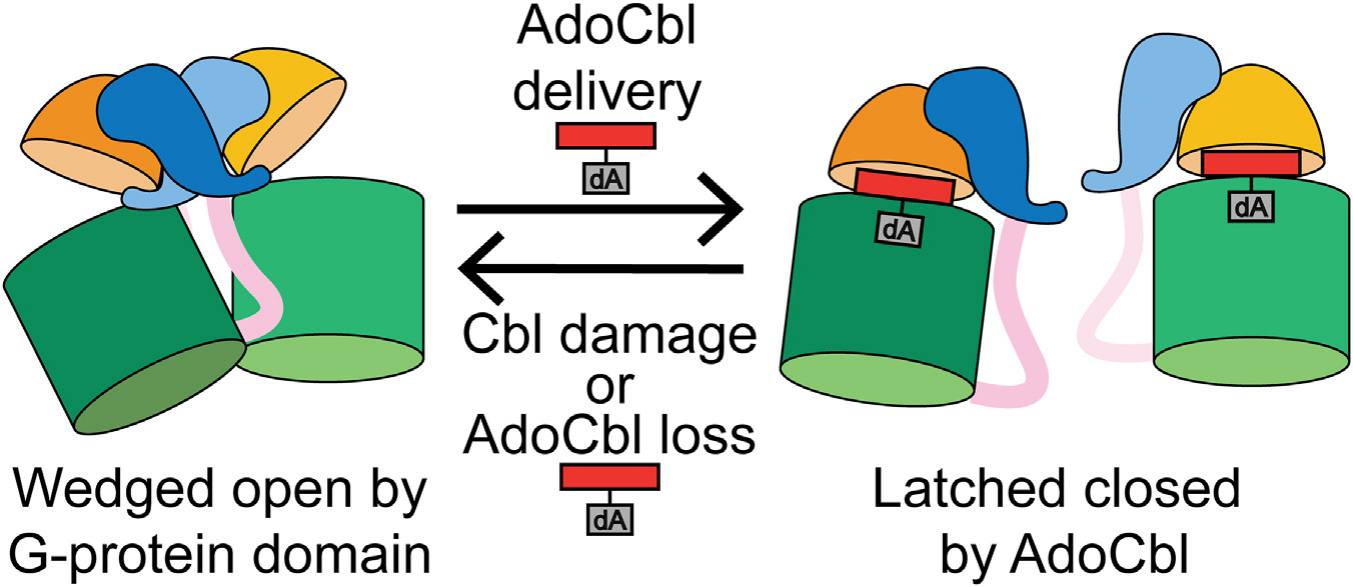
**Cartoon depicting equilibrium between open and closed states of IcmF mutase.** The equilibrium is expected to shift to the *right* in the presence of a bound AdoCbl due to the adenosyl moiety of AdoCbl, which juts into the substrate-binding domain (*green*), securing the Cbl-binding domain (*orange*) against the substrate-binding domain. The equilibrium is expected to shift to the *left* in the case of AdoCbl damage or loss. Without the adenosyl moiety jutting across the mutase interface, the open conformation is more favorable, which in turn would facilitate IcmF oligomerization as the open mutase conformation has a preformed binding pocket for the G-protein “wedge.” The second protomer of each IcmF molecule is not shown for simplicity. AdoCbl, adenosylcobalamin; Cbl, cobalamin; IcmF, isobutyryl-CoA mutase fused.

Chemical logic dictates that a metallochaperone delivery/repair system should only open a target enzyme for cofactor delivery in the apo-state of enzyme and/or following cofactor damage, as it is wasteful to replace a working cofactor. The structure of IcmF suggests that the Cbl itself may regulate the open/closed equilibrium of the mutase (Fig. 8). In particular, IcmF structures show that the adenosyl moiety of AdoCbl reaches across the boundary between the Cbl-binding domain to the substrate-binding domain, thereby securing the domains together (29) and shifting the conformational equilibrium toward a closed mutase state. Loss of the adenosyl moiety due to photolysis or oxidative damage is expected to loosen the connection between domains, facilitating the movement of the Cbl-binding domain and shifting the conformational equilibrium toward the open mutase state (25, 26, 27, 29). The cryo-EM structure presented here shows that an open conformation of IcmF creates a pocket that can be filled by a neighboring IcmF’s G-protein domain and, in particular, its wedge helix (purple residues 363–380) and its switch III region (Fig. 7*D*). Thus, loss of AdoCbl or damage to AdoCbl should shift the conformational equilibrium of IcmF to an open state, facilitating the binding of a G-domain of a neighboring IcmF molecule (Fig. 8) and starting the repair process. In addition to data showing that switch III residues play a role in the removal of damaged Cbl (31), our negative stain EM data using GMPPNP and GMPPCP show more extensive IcmF oligomerization when AdoCbl is subjected to photolysis or replaced with OHCbl than for intact AdoCbl (Fig. 5, *C* and *D*). We expect that *in vivo*, the conformational equilibrium shifts toward the open mutase structure due to Cbl damage or AdoCbl loss will be necessary for oligomerization, limiting the wasteful replacement of a working cofactor. In terms of maturation, the formation of the supramolecular complexes made up of apo-IcmF molecules with GMPPCP (Fig. 4*D*, right) invokes an assembly line model in which a chain of open mutases can be filled in succession by an ATR that moves down the chain, delivering AdoCbl as it is synthesized. Future experiments aimed at establishing the length of apo-IcmF complexes *in vivo* would provide insight into this hypothesis. We currently do not know if IcmF forms an oligomer longer than a tetramer *in vivo* nor do we know how ATR interacts with IcmF. A structure of these IcmF supramolecular complexes or of an IcmF tetramer in the presence of ATR would be highly valuable.

Collectively, the structural and biochemical data obtained previously (6, 29, 32, 33) and presented here indicate that the only significant difference between the standalone system (Fig. 2*A*) and the fused IcmF (Fig. 2*B*) is whether the active G-protein state is formed *via* a conformational change or *via* oligomerization. It is not unprecedented for signaling proteins to employ both methods (conformational change and oligomerization) to switch between active and inactive states. For example, members of the CAP family of transcription factors can use either ligand binding to induce dimerization that affords DNA association or ligand binding to induce a conformational change that affords DNA association (38, 39). The use of two different methods for creating an active G-protein state makes sense if one considers that G-protein chaperones are designed to be poor GTPases in the absence of their target protein to prevent unwanted GTP hydrolysis. Since the G-protein and target protein are fused in IcmF, unwanted GTP hydrolysis would be a large problem if the active G-protein state was easily formed. For MeaB, MCM is its GAP. For the G-protein domain of IcmF, however, a second IcmF protomer is the GAP, and our cryo-EM data show that residues required for GTP hydrolysis (K344 for example) are on the second protomer (Fig. 6). Thus, the active site for GTP hydrolysis is missing in IcmF’s resting homodimeric state, which is the state of wt IcmF that is visible in the absence of GTP or GTP analogs (Fig. 4). If the G-protein domains of IcmF were present as an obligate dimer, allowing a conformational change to create an active site capable of GTP hydrolysis, chemical logic suggests that the unwanted GTP hydrolysis would be a more substantial problem. IcmF is a poor GTPase because its active site residues are not part of its resting structure.

The presence of fused systems has not been reported for other members of the SIMIBI class of P-loop G-proteins, such as UreG and HypE, which also rely on binding a G-nucleotide to form the active conformation of the G-protein. Instead, UreG and HypE resemble MeaB (40); however, the degree to which these metallochaperones utilize the same molecular mechanisms is unclear. Each metallochaperone system appears to have a different number of accessory proteins, which are commonly of unknown function and unknown structure, and if structures exist, the structures are often of inactive states or isolated states not of the protein:protein complex that is responsible for maturation. Our results here suggest that cryo-EM is likely to be crucial for obtaining structures of transient protein complexes, affording new snapshots of the metallochaperone delivery and repair processes. We hope that the studies presented here will benefit metalloenzyme applications in industry and in medicine by providing structural and mechanistic insight into one system in detail. With the cryo-EM resolution revolution, we expect that this work is the beginning of what will be an exciting decade for this field.

## Experimental procedures

All chemicals, solvents, and reagents were purchased from Sigma-Aldrich unless otherwise noted.

### Plasmids

**Table:** 

Name	Features	Source
pET28a_*cm*IcmF	N-terminal His-tag, thrombin cleavage site, T7 protomer, Kan^R^	Ref. (29)
pET28a_*cm*IcmF_Q341A	N-terminal His-tag, thrombin cleavage site, T7 protomer, Kan^R^	This study
pET28a_*cm*IcmF_K344A	N-terminal His-tag, thrombin cleavage site, T7 protomer, Kan^R^	This study

### Cloning

The plasmid containing the WT *C. metallidurans icmF* gene as described previously (29) was used as the template for site-directed mutagenesis. Site-directed variants were generated using a Quikchange II XL site-directed mutagenesis kit (Agilent) using the following primers.

**Table:** 

Name	Sequence (5′ to 3′)
IcmF_Q341A_F	GCGCGGCCAGCGCGCTCGAGAAGATCGAC
IcmF_Q341A_R	GTCGATCTTCTCGAGCGCGCTGGCCGCGC
IcmF_K344A_F	GCGAGCCAGCTCGAGGCGATCGACATGCTCGACTTCGC
IcmF_ K344A_R	GCGAAGTCGAGCATGTCGATCGCCTCGAGCTGGCTGGC

Mutations generated according to the Quikchange protocol were confirmed by Sanger sequencing (Genewiz) using the following primers for full sequencing overlap.

**Table:** 

Name	Direction	Sequence (5′ to 3′)
IcmF_seq_1	Forward	GCGCAACTGATTACCGCG
IcmF_seq_2	Forward	CAAGCAGGTGCAGCGCAA
IcmF_seq_3	Forward	CGTGTTCGCGTTCAAGCG
IcmF_seq_4	Forward	GAAGCCGGTGCGAATCCG
IcmF_seq_5	Reverse	CCACATCGCCAGCAGCTTG

### Protein expression and purification

Cell growth and purifications of wt IcmF, Q341A IcmF, and K344A IcmF from *C. metallidurans* were conducted following the same procedure described here. An overnight culture of 100 ml of lysogeny broth medium (Fisher BioReagents) supplemented with 50 μg/L kanamycin (GoldBio) was inoculated from a single colony of *Escherichia coli* BL21 T7 Express competent cells (New England Biolabs) transformed with the appropriate gene and grown at 37 °C with shaking. The overnight starter culture was used to inoculate 1 L of lysogeny broth supplemented with 50 μg/L kanamycin at 37 °C. The 1 L culture was placed at 16 °C with shaking when A_600_ reached ∼0.5 to 0.6. After 2 h, the 1 L culture was induced with a final concentration of 0.1 mM IPTG (GoldBio) and grown for 10 h to 12 h at 16 °C with shaking. Cells were harvested by centrifugation (5000*g*, 4 °C, 20 min) and flash frozen in liquid N_2_ before being stored in a −80 °C freezer for future use.

Cells from 2 L of cell culture were resuspended in 80 mL of lysis buffer (50 mM Hepes pH 7.5, 500 mM NaCl, 20 mM imidazole) supplemented with one cOmplete EDTA-free protease inhibitor tablet (Roche), 1 mM of PMSF, 1 mM of benzamidine HCl, and benzonase nuclease. Cells were lysed by ultrasonification, and cell lysates were clarified by centrifugation (28,000*g*, 30 min, 4 °C). Clarified lysate was passed through a 0.2 μm filter before being loaded onto a 5 ml Ni-NTA column (GE Healthcare) equilibrated with lysis buffer using an fast protein liquid chromatography (FPLC) system (BioRad NGC System). The column was washed with 10 column volumes of lysis buffer and 10 column volumes of 50 mM Hepes pH 7.5, 500 mM NaCl, and 40 mM imidazole using an FPLC. Protein was eluted with 50 mM Hepes pH 7.5, 500 mM NaCl, 200 mM imidazole using an FPLC with a flow rate of 4 mL/min. Elution fractions were buffer exchanged into 50 mM Hepes pH 7.5 and then concentrated in a 50 kDa MWCO centrifugal filter. The concentrated fractions were loaded onto a MonoQ 10/100 anion exchange chromatography column (Cytiva) prepped with 50 mM Hepes pH 7.5 and 5 mM NaCl. Protein was eluted with a linear gradient from 10% to 80% of 50 mM Hepes pH 7.5 and 500 mM NaCl with a flow rate of 2 mL/min. The protein eluted in a sharp peak at around 250 mM to 300 mM NaCl. There was a second peak immediately following the first peak, corresponding to aggregated/truncated protein. The eluted protein was concentrated in a 50 kDa MWCO centrifugal filter. The concentrated fractions of full-length IcmF were loaded onto a Superdex 200 16/60 size-exclusion chromatography (SEC) column (GE Healthcare) equilibrated with SEC buffer (20 mM Hepes pH 8, 50 mM NaCl) and eluted with a flow rate of 1 mL/min. Elution fractions from SEC were concentrated in a 50 kDa MWCO centrifugal filter. Purity was assessed by a 4 to 20% (w/v) SDS-PAGE (Bio-Rad). The concentration of the IcmF monomer was determined by UV/Vis absorbance at 280 nm using an extinction coefficient of 84,600 M^−1^ cm^−1^, determined using the ProtParam tool (41). Protein samples at a concentration of ∼10 mg/mL (80 μM) in SEC buffer were flash frozen in liquid N_2_ and stored in a −80 °C freezer for future use.

### GTPase assays

The GTPase activity of wt IcmF, Q341A IcmF, and IcmF K344A was determined in the presence of various concentrations of GTP (2–1500 μM) (Roche). The EnzChek phosphate assay kit (Molecular Probes) was used for all GTPase assays following the manufacturer’s instructions with the following modifications. The assay reactions (200 μL) were prepared excluding the enzyme (wt IcmF, Q341A IcmF, K344A IcmF, or no enzyme SEC buffer control) and incubated at room temperature (23 °C) for 5 min before initiating the assay to control for contaminating inorganic phosphate in the GTP. After incubation, the assays were initiated with the addition of 0.5 μM enzyme or 10 μL of IcmF SEC buffer. After 7 s of initial mixing, the absorbance at 360 nm for each assay reaction was recorded every 8 s with 2 s of mixing in between readings for 15 min total using a SpectraMax Plus 384 microplate reader (Molecular Dimensions). The absorbances were converted into concentration of inorganic phosphate using the standard curve generated according to the manufacturer’s directions. The initial rates were calculated for each assay reaction and subtracted from the average initial rates of the no enzyme control of the corresponding concentration of GTP. The Michaelis–Menten parameters (*k*_*cat*_, *K*_*m*_, and *V*_*max*_) were generated using a nonlinear regression by Prism v9.4.1 (Graphpad). Reported values ± SD are the results of at least three independent experiments (Table 1).

### Mass photometry

Mass photometry (interferometric scattering mass spectrometry) was performed on a Refeyn instrument using AcquireMP v2.4.1 and DiscoverMP v2.4.2. All movies were taken for a length of 60 s using the default parameters. The contrasts were converted into molecular weights using the standard curve generated from a sample of NativeMark Unstained Protein Standard (Novex by life Technologies). Gaussian curves were fit to each histogram distribution, and the mass (kDa), sigma (kDa), and normalized counts were determined using the PhotoMol software (https://spc.embl-hamburg.de/app/photoMol) (42). Each percentage of total counts ± SD reported are the results of at least three independent experiments. All samples that contained any additives also contained 500 μM MgCl_2_. Samples were incubated with 500 μM GDP or no nucleotide for 15 min on ice at a concentration of 80 ng/μl prior to the final dilution to 8 ng/μL on the instrument and data recording. Samples were incubated with 500 μM GMPPCP for 15 min on ice at a concentration of 160 ng/μl prior to the final dilution to 16 ng/μL on the instrument and data recording. Samples containing 500 μM GTP were initially diluted to 160 ng/μL before the final dilution to 16 ng/μL in SEC buffer (20 mM Hepes pH 8, 50 mM NaCl) supplemented with 500 μM GTP on the instrument and data recording. Samples containing the substrate butyryl-CoA (500 μM) and/or various nucleotides (GDP or GMPPCP) were incubated for 15 min on ice at a concentration of 200 ng/μL prior to the final dilution to 20 ng/μL on the instrument and data recording. Reported values ± SD are the results of at least three independent experiments.

### Negative stain EM specimen preparation and imaging

wt IcmF, Q341A IcmF, or K344A IcmF was thawed on ice and diluted to 20 ng/μL in IcmF SEC buffer (20 mM Hepes pH 8, 50 mM NaCl). Each of the samples except samples with GTP were incubated for 30 min on ice with the corresponding nucleotide (GDP or GMPPCP) before grid preparation. The final concentration of any additives (nucleotides: GDP, GTP, or GMPPCP; cofactor: AdoCbl or OHCbl; MgCl_2_) in the samples were 500 μM. For the samples containing GTP, the GTP was added, and the protein solution immediately applied to the grid. For the samples containing AdoCbl and exposed to light, after incubation for 30 min in the dark, the protein solution was then exposed to a white light for 15 min before application on the grid.

Carbon-coated 300 mesh copper EM grids (Electron Microscopy Services) were glow discharged for 1 min at −15 mA. An aliquot (5 μl) of the protein solution was applied to the grid; after approximately 1 min, the solution was blotted and immediately replaced with solution of 2% uranyl acetate (VWR). The stain solution was blotted and replaced twice, then allowed to stand for 1 min before the final blot, and then was dried. All blotting was done manually using filter paper (Whatman, grade 40). The specimens were imaged with an AMT Nanosprint5 camera on a FEI Morgagni electron microscope operated at 80 kV. Images were collected at 18,000× magnification.

### Cryo-EM grid preparation

The grids used to investigate the supramolecular complex of IcmF in the presence of GMPPCP (wt IcmF + GMPPCP) were prepared as follows: 0.2 mg/mL graphene oxide suspension was prepared using molecular grade water. The suspension was centrifuged at 300*g* for 1 min to remove large aggregates. A Quantifoil 1.2 to 1.3 Cu 300 mesh holey-carbon grid (Electron Microscopy Services) was glow discharged at −40 mA at 0.1 bar for 2 min before application of 3 μL of the graphene oxide suspension. The suspension was incubated for 1 min before excess suspension was blotted away (Whatman, grade 40). The grid was washed twice on the graphene oxide suspension side and once on the backside of the grid in molecular grade water and dried. The graphene oxide–covered grid was plunged on a Thermo Fisher Scientific Vitrobot (Mk IV) cryo-plunger. The final protein solution contained 2 μM wt IcmF, 500 μM GMPPCP, and 500 μM butyryl-CoA incubated in SEC buffer for at least 15 min before application. The sample (5 μL) was applied to the grids that were blotted for 5 s with a blot force of 10 (Whatman filter paper #1) before plunging into liquid ethane and transferring to storage grids. The temperature and humidity inside the Vitrobot chamber were set to 8 °C and 95%, respectively.

The grids used to investigate the conformation of Q341A IcmF in the presence of GTP (Q341A IcmF + GTP) were prepared as follows: a Quantifoil R 1.2 to 1.3 Cu 300 mesh holey-carbon grid (Electron Microscopy Services) was glow discharged at −15 mA at 0.039 bar for 1 min before application of the protein solution. The final protein solution contained 2 μM Q341A IcmF, 500 μM GTP in SEC buffer. The sample (5 µL was applied to the grids that were blotted for 3 s with a blot force of 10 (Whatman filter paper #1) before plunging into liquid ethane and transferring to storage grids. The temperature and humidity inside the Vitrobot chamber were set to 8 °C and 95%, respectively.

### Cryo-EM data collection

Data were collected at the MIT.nano Center for Automated Cryogenic Electron Microscopy at the Massachusetts Institute of Technology on an FEI Talos Arctica G2 Cryo 200 kV transmission electron microscope equipped with a Falcon 3EC camera. The data collection parameters for the IcmF + GMPPCP grid were as follows: 73,000× magnification resulting in a pixel size of 2.043 Å, 14 frames, 10.5 e^-^/Å^2^/frame dose, and defocus range 1.2 to 3.1 μm. The dataset contained 602 movies. The data collection parameters for the Q341A IcmF + GTP grid were as follows: 92,000× magnification resulting in a pixel size of 1.5998 Å, 14 frames, 14.7 e^-^/Å^2^/frame dose, and defocus range 1.2 to 3.1 μm. The dataset contained 673 movies. These parameters are summarized in Table S1.

### Cryo-EM data processing and model refinement

Cryo-EM data processing of the datasets was carried out using a combination of Relion 4.0-beta (43) and CryoSPARC v3.3.2 (36) and is summarized in Fig. S4. For the wt IcmF + GMPPCP dataset, individual frames of dose-fractionated exposures were aligned and summed using Relion’s implementation of MotionCor2 (44) and the defocus of the summed frames was estimated using Relion’s implementation of CTFFind4 (45). The start-end coordinates for the locations of the helical supramolecular complexes were manually determined for all micrographs. Using these coordinates, 510,941 particles were extracted using a box size of 128 pixels (257.8 Å), tube diameter of 200 Å, one asymmetric unit, and a helical rise of 10 Å (Fig. S4). These particles were imported in CryoSPARC for the rest of the data processing. The particles were subjected to one round of initial reference-free 2D classification with a mask of 200 Å to generate 50 class averages. After removing the classes that visually did not look like particles, another round of reference-free 2D classification was performed with the remaining 459,669 particles to generate 50 2D class averages. The 439,589 particles selected after the second round of 2D classification were used to generate two *ab initio* initial reference-free models using no imposed symmetry. The initial models were subjected to heterogenous refinement. The class consisting of 363,613 intact particles was subjected to homogenous refinement. After homogenous refinement, the aligned particles were subjected to a local refinement using CryoSPARC’s new implementation. These aligned particles were checked for duplicate particles. The remaining 165,181 aligned particles were subjected to signal subtraction to remove signal from the ends of the supramolecular complexes that were artifacts of processing helical data as single particles using a manually generated mask. These subtracted particles were then subjected to a local refinement using CryoSPARC’s new implementation. These aligned particles were then checked for duplicate particles. The final 138,956 particles were subject to one more round of local refinement. CryoSPARC’s dynamic masking was utilized for all refining steps, excluding the signal subtraction. Combination of the two half-maps along with local B-factor adjustment was performed on the COSMIC^2^ server’s implementation (46) of LocSpiral (37) with a low pass filter of 15 Å, bandwidth of band pass filter of 8 Å, and an initial binarization threshold of 0.679. The Fourier shell correlation (FSC) plots were generated by CryoSPARC. The final masked resolution at FSC = 0.143 was 6.7 Å (Fig. S9*A*).

For the Q341A IcmF + GTP dataset, all data processing was performed in CryoSPARC v3.3.2 (36) and is summarized in Fig. S5. First, individual frames of dose-fractionated exposures were aligned and summed using patch motion correction. Next, the defocus of the summed frames was estimated using CryoSPARC’s patch contrast transfer function estimation. To generate the coordinates of the particles, the blob picker was used with minimum particle diameter of 200 Å and maximum particle diameter of 350 Å. Using a normalized cross-correlation score above 0.160 and local power between −8,352 and 242,864, 180,271 particles were extracted with a box size of 196 pixels. These particles were subjected to one round of initial reference-free 2D classification to generate 200 2D class averages. After removing classes that visually did not look like particles, another round of reference-free 2D classification was performed with the remaining 74,767 particles to generate 100 2D class averages. The 70,582 particles selected after the second round of 2D classification were used to generate three *ab initio* initial reference-free models using no imposed symmetry. For the following heterogenous refinement, two of the *ab initio* models (one containing intact complex and one containing junk particles) were supplied. Only the 59,335 particles associated with the model containing intact complex were subject to a homogenous refinement. These particles were then subjected to a local refinement using CryoSPARC. CryoSPARC’s dynamic masking was utilized for all refining steps. Combination of the two half-maps along with local B-factor adjustment was performed on the COSMIC^2^ server’s implementation (46) of LocSpiral (37) with a low pass filter of 15 Å, bandwidth of band pass filter of 8 Å, and an initial binarization threshold of 0.878. The FSC plots were generated by CryoSPARC. The final masked resolution at FSC = 0.143 was 4.6 Å (Fig. S9*B*).

For model building and refinement, one protomer of the dimeric IcmF (chain B from PDB 4XC6, residues 22–1093) was segmented into two fragments that were manually docked into the maps resulting from the reconstruction of the wt IcmF + GMPPCP and Q341A IcmF + GTP datasets, respectively (29). All the ligands and water molecules were removed from the fragments. Despite the low to modest resolution, side chains were retained in the fragments. The first fragment consisted of the Cbl-binding domain and the G-protein domain (residues 22–442); the second fragment consisted of substrate-binding domain and the linker region (residues 443–1093). For the wt IcmF + GMPPCP reconstruction, four complete protomers (eight fragments) of IcmF were manually docked in the map in ChimeraX v1.4 (47). Using Phenix real-space refinement, the resulting model was subjected to one round of refinement consisting of rigid body refinement, with each fragment refined as an individual rigid body. There was no clear density for substrate or GMPPCP and, therefore, these ligands were not modeled in. For the Q341A IcmF + GTP reconstruction, three copies of a complete protomer of IcmF (six fragments) were manually docked into the map in ChimeraX v1.4. Although the map appeared to contain two IcmF homodimers, the density was only good enough to place three protomers (six fragments) and no atoms were modeled into a fourth protomer. Within the modeled protomers, however, there was density present for residues 1,011 to 1,018, which were previously disordered in the crystal structures, and those residues were added manually by model building in Coot (48). Additionally, there was clear density for GDP at each nucleotide-binding site. In the nucleotide-binding site for chain A, there was enough density to model in a magnesium ion in addition to the GDP. Using Phenix real-space refinement (49), the docked models were subjected to one round of refinement consisting first of simulated annealing and then rigid body refinement, with each copy of each fragment of IcmF defined as an individual rigid body. The second round of real-space refinement was carried out on the model with rigid body refinement and minimization. Molecular building and refining software packages were compiled by SBGrid (50).

## Data availability

Atomic coordinates have been deposited in the Protein Data Bank under accession codes 8SSL and 8STA. The cryo-EM density maps have been deposited in the Electron Microscopy Data Bank under accession number EMD-40751 and EMD-40758.

## Supporting information

This article contains supporting information (51, 52).

## Conflict of interest

C. L. D. is a Howard Hughes Medical Investigator. M. J. consults for Gate Biosciences and Evozyne. The other authors declare that they have no conflicts of interest with the contents of this article.
