# Supplementary material for: Structural insight into G-protein chaperone-mediated maturation of a bacterial adenosylcobalamin-dependent mutase
Source: J Biol Chem. 2023 Jul 28;299(9):105109. doi: 10.1016/j.jbc.2023.105109 (PMC10481361; doi:10.1016/j.jbc.2023.105109)
Supplement: Supporting Figures S1–S9 and Table S1 [file mmc1.pdf]

Supporting information for

Structural insight into G-protein chaperone-mediated maturation of a bacterial adenosylcobalamin-dependent mutase

Francesca A. Vaccaro[1], Daphne A. Faber[2], Gisele A. Andree[1], David A. Born[3,4,a], Gyunghoon Kang[1,b], Dallas R. Fonseca[5,c], Marco Jost[1,d], and Catherine L. Drennan[1,4,6*]

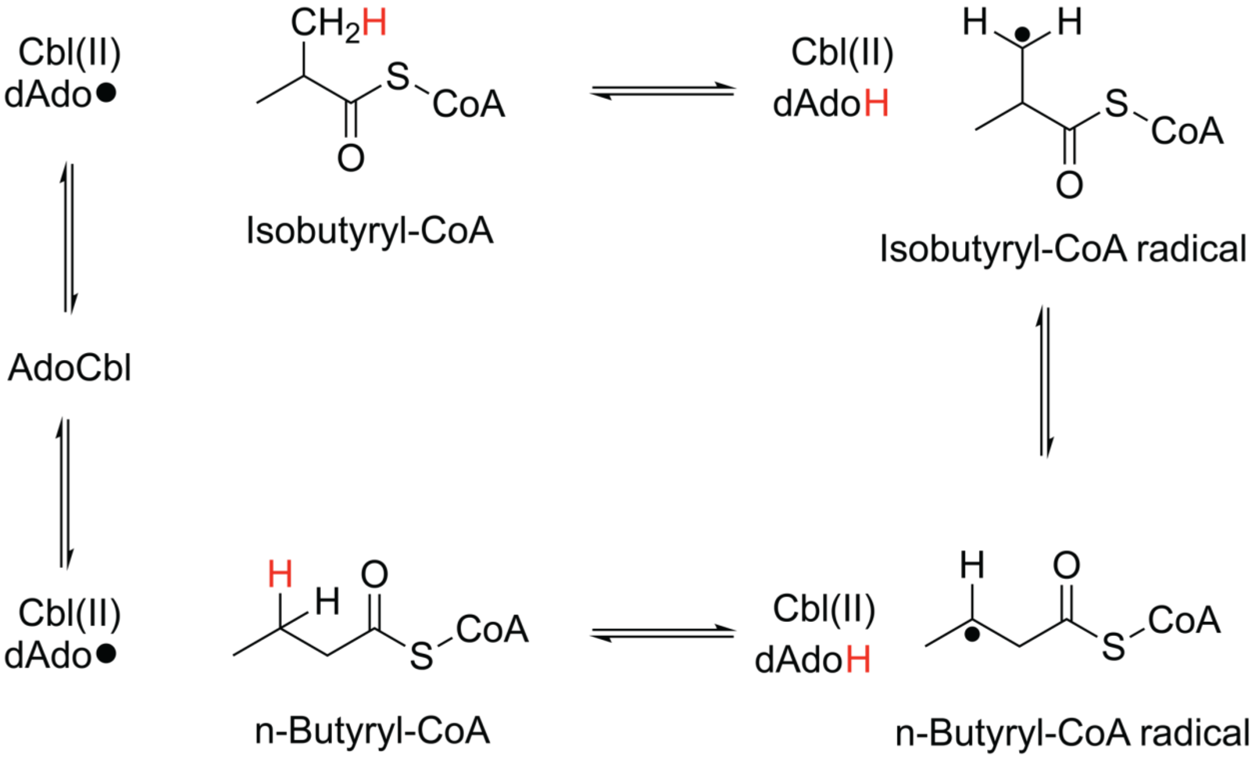

Figure S1. Scheme showing proposed catalytic mechanism for IcmF. The rearrangement of isobutyryl-CoA to *n*-butyryl-CoA requires the formation of a 5′-deoxyadenosyl radical (dAdo•) from the homolytic cleavage of AdoCbl to perform a hydrogen atom abstraction from substrate. The product *n*-butyryl-CoA radical re-abstracts the hydrogen atom from 5′-deoxyadenosine (dAdoH), regenerating dAdo•. Cob(II)alamin (Cbl(II)) and dAdo• reform AdoCbl for the next round of turnover (51).

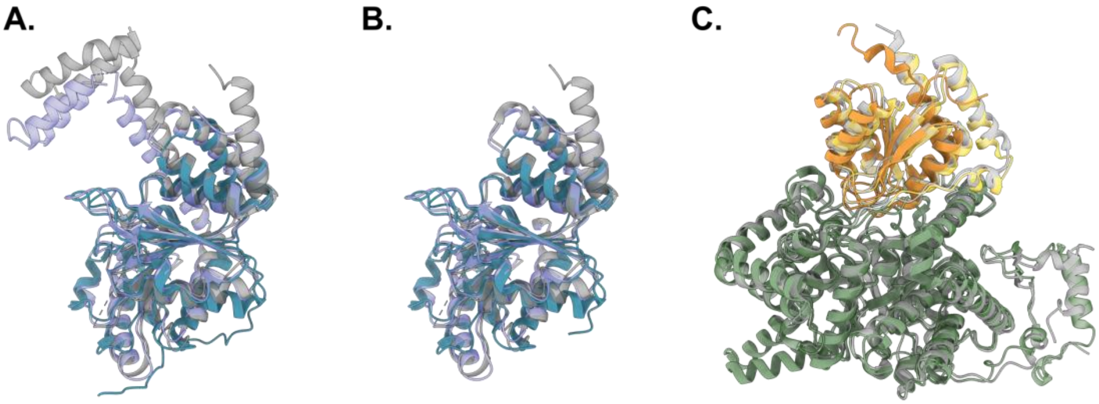

Figure S2. The domains of IcmF are similar to the corresponding two component analogs. **A**. The G-protein domain of IcmF (PDB 4XC6) (29) (blue ribbons) overlayed with one protomer of MeaB (RMSD for C∝: 0.956 Å) (PDB 8DPB) (33) (purple ribbons) and one protomer of MMAA (RMSD for C∝: 1.065 Å) (PDB 2WWW) (6) (grey ribbons). **B**. The same overlay in (A) with the C-terminal dimerization helices of MeaB and MMAA removed to highlight the similarity of the core G-domain. **C.** The Cbl-binding domain (orange ribbons) and substrate binding domain (green ribbons) of IcmF (PDB 4XC6) (29) overlayed with the active subunit (Cbl-binding domain: yellow; substrate-binding domain: dark green) of methylmalonyl-CoA mutase from *P. shermanii* (RMSD for C∝: 1.209 Å) (PDB 4REQ) (26) and one protomer of methylmalonyl-CoA mutase from *H. sapiens* (RMSD for C∝: 0.948 Å) (PDB 2XIQ) (6) (grey ribbons).

```
CmIcmF        ------------------------------------------------------------      168
MeMeaB        ------------------------------------------------------------      0
HsMMAA        MPMLLPHPHQHFLKGLLRAPFRCYHFIFHSSTHLGSGIPCAQPFNSLGLHCTKWMLLSDG      60

CmIcmF        -----------------------------LDTVVAGDRRAA-QLITALENGKADPE----      195
MeMeaB        ------------------MSATLPDMDTLRERLLAGERAALARAITLAESRRADHRAAVR      42
HsMMAA        LKRKLCVQTTLKDHTEGLSDKEQRFVDKLYTGLIQGQRACLAEAITLVESTHSRKKELAQ      120
                                              :: *:* .  . **  *. ::  .

CmIcmF        -LVSAL---H-----AQAKAAAVPVLGITGTGGAGKSSLTDELIRRFRLDQDDALSIAVI      246
MeMeaB        DLIDAVL----------PQTGRAIRVGITGVPGVGKSTTIDALGSLL---TAAGHKVAVL      89
HsMMAA        VLLQKVLLYHREQEQSNKGKPLAFRVGLSGPPGAGKSTFIEYFGKML---TERGHKLSVL      177
               *:. :                .  :*::*  *.***:  : :   :      . .::*:

CmIcmF        SIDPSRRKSGGALLGDRIRMNAIN-HPNIFMRSLATREAGSEISQALPDVIAACKAARFD      305
MeMeaB        AVDPSSTRTGGSILGDKTRMARLAIDRNAFIRPSPSSGTLGGVAAKTRETMLLCEAAGFD      149
HsMMAA        AVDPSSCTSGGSLLGDKTRMTELSRDMNAYIRPSPTRGTLGGVTRTTNEAILLCEGAGYD      237
              ::***   :**::***: **  :  . * ::*   :  : . ::    :.:  *:.* :*
                                          Switch III
CmIcmF        LVIVETSGIGQGDAAIVPHVDLSLYVMTPEFGAASQLEKIDMLDFADFVAINKFDRKGA-      364
MeMeaB        VILVETVGVGQSETAVADLTDFFLVLMLPGAGDELQGIKKGILELADMIAVNKADDGDGE      209
HsMMAA        IILIETVGVGQSEFAVADMVDMFVLLLPPAGGDELQGIKRGIIEMADLVAVTKSD-GDLI      296
              :::**  *:**.: *:.  .*: : :: *  *   *  * .::::**::*:.* *  .

CmIcmF        ------QDAWRDVAKQVQRNREQWHSRAEDMPVYGTQASRFNDDGVTMLYQGLVGALGAR      418
MeMeaB        RRANAAASEYRAALHILTPPSATWTPPVVTIS-------GLHGKGLDSLWSRIEDH---R      259
HsMMAA        VPARRIQAEYVSALKLLRKRSQVWKPKVIRIS-------ARSGEGISEMWDKMKDF---Q      346
                       :  . : :      *   .  :           ..*:  ::. : .    :

CmIcmF        GMSLKPGTL---------------------------------PNLEGRISTGQNV----      440
MeMeaB        AKLTATGEIAGKRREQDVKWMWALVHERLHQRLVGSAEVRQATAEAERAVAGGEHS----      315
HsMMAA        DLMLASGELTAKRRKQQKVWMWNLIQESVLEHFRTHPTVREQIPLLEQKVLIGALSPGLA      406
                    * :                                     *  :  *

CmIcmF        -IV---------      442
MeMeaB        ------------      315
HsMMAA        ADFLLKAFKSRD      418
```

Figure S3. Sequence alignment of the G-protein domain of IcmF from C. metallidurans with MeMeaB and HsMMAA. The switch III region which participates in binding and catalysis of the nucleotide substrate is highlighted. The conserved residues of the switch III region that were substituted to alanine are bolded. Asterisks (*) denote positions with conserved residues. Colons (:) denote positions with conservation with strongly similar properties. Periods (.) denote positions with conservation with weakly similar properties. Alignments performed using Clustal Omega (52).

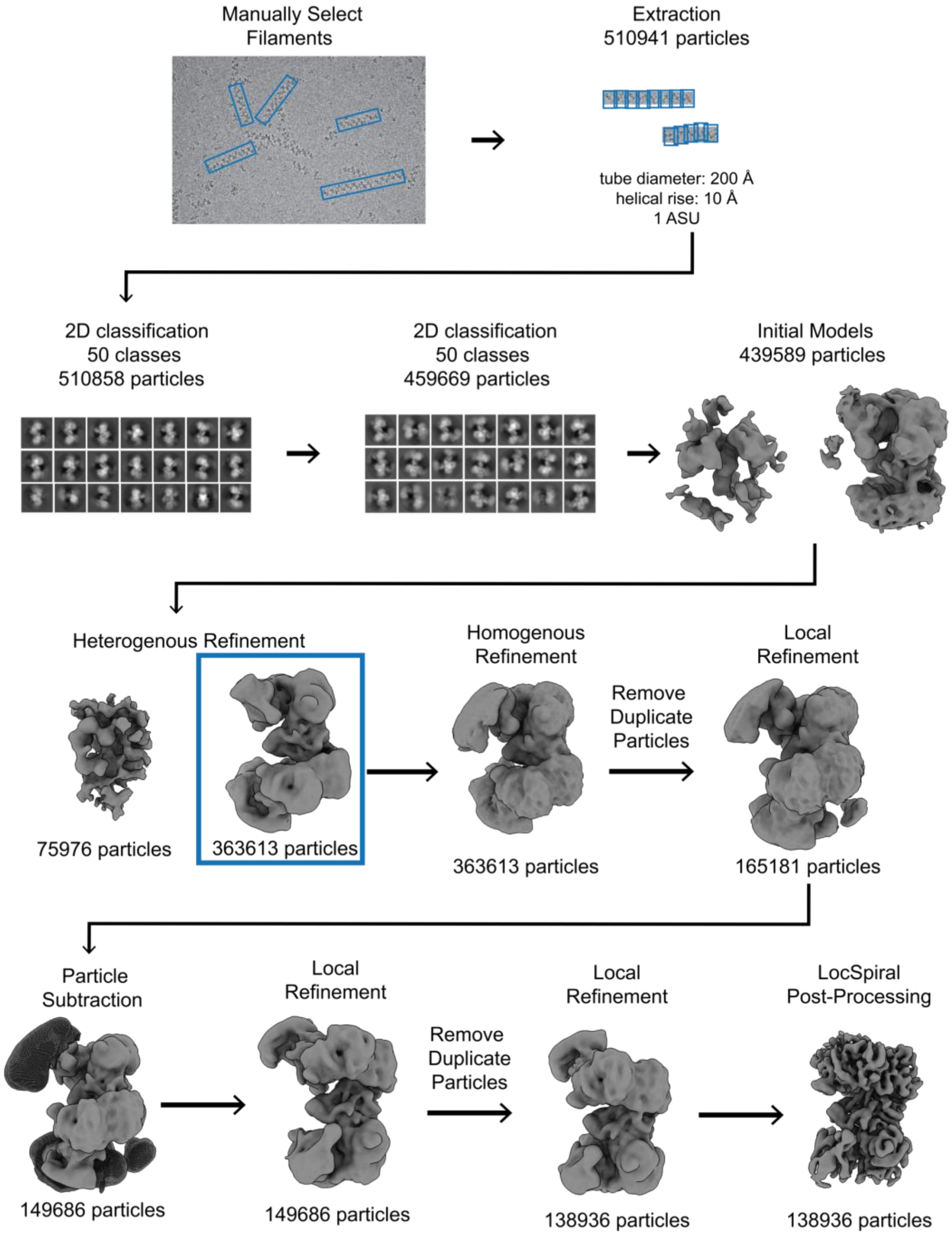

Figure S4. Data processing workflow for the dataset of wt IcmF in the presence of GMPPCP and butyryl-CoA. Drift assessment and initial contrast transfer function (CTF) assessment was performed, filaments were manually traced, and particles were extracted in Relion 4.0. All subsequent analyses except for post-processing B-factor sharpening, which was performed using COSMIC[2]'s implementation of LocSpiral, was performed using CryoSPARC.

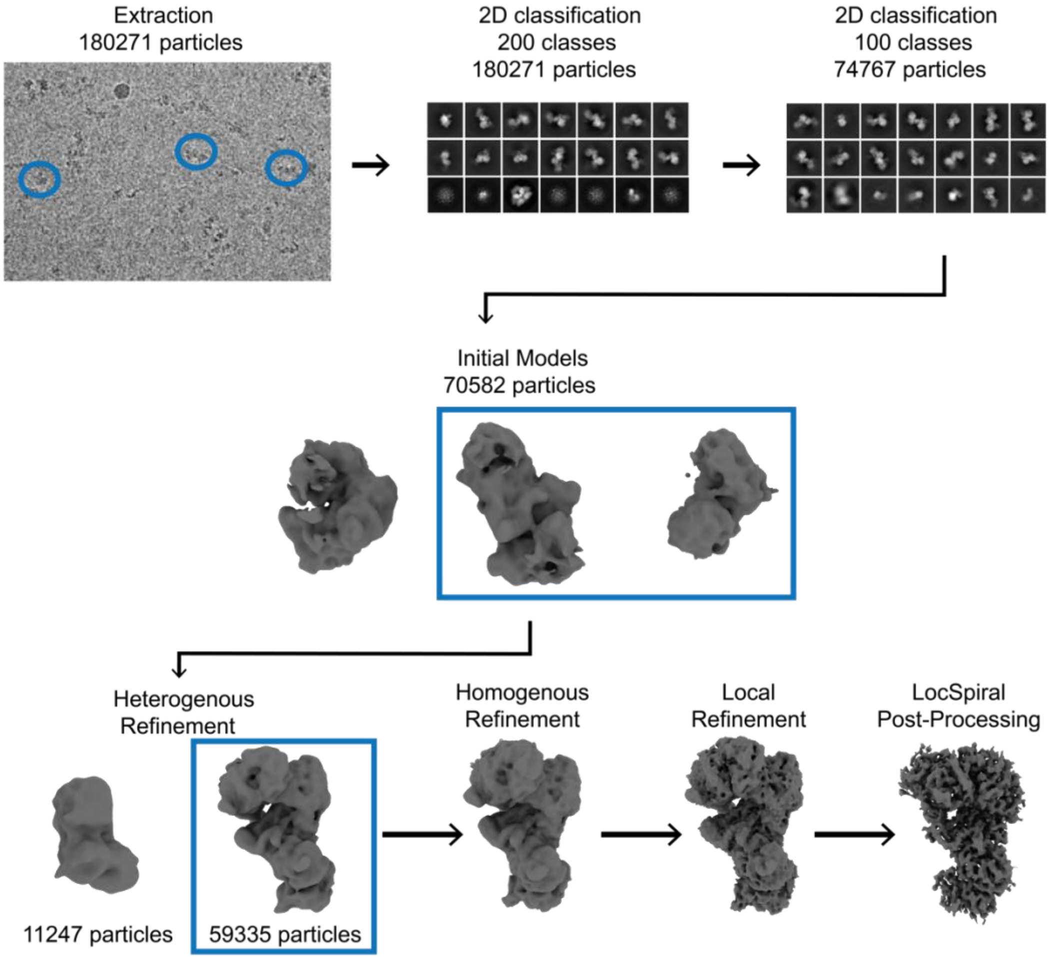

Figure S5 The processing workflow for the dataset of the Q341A IcmF variant in the presence of GTP. All analyses except for post-processing B-factor sharpening, which was performed using COSMIC[2]'s implementation of LocSpiral, was performed using CryoSPARC.

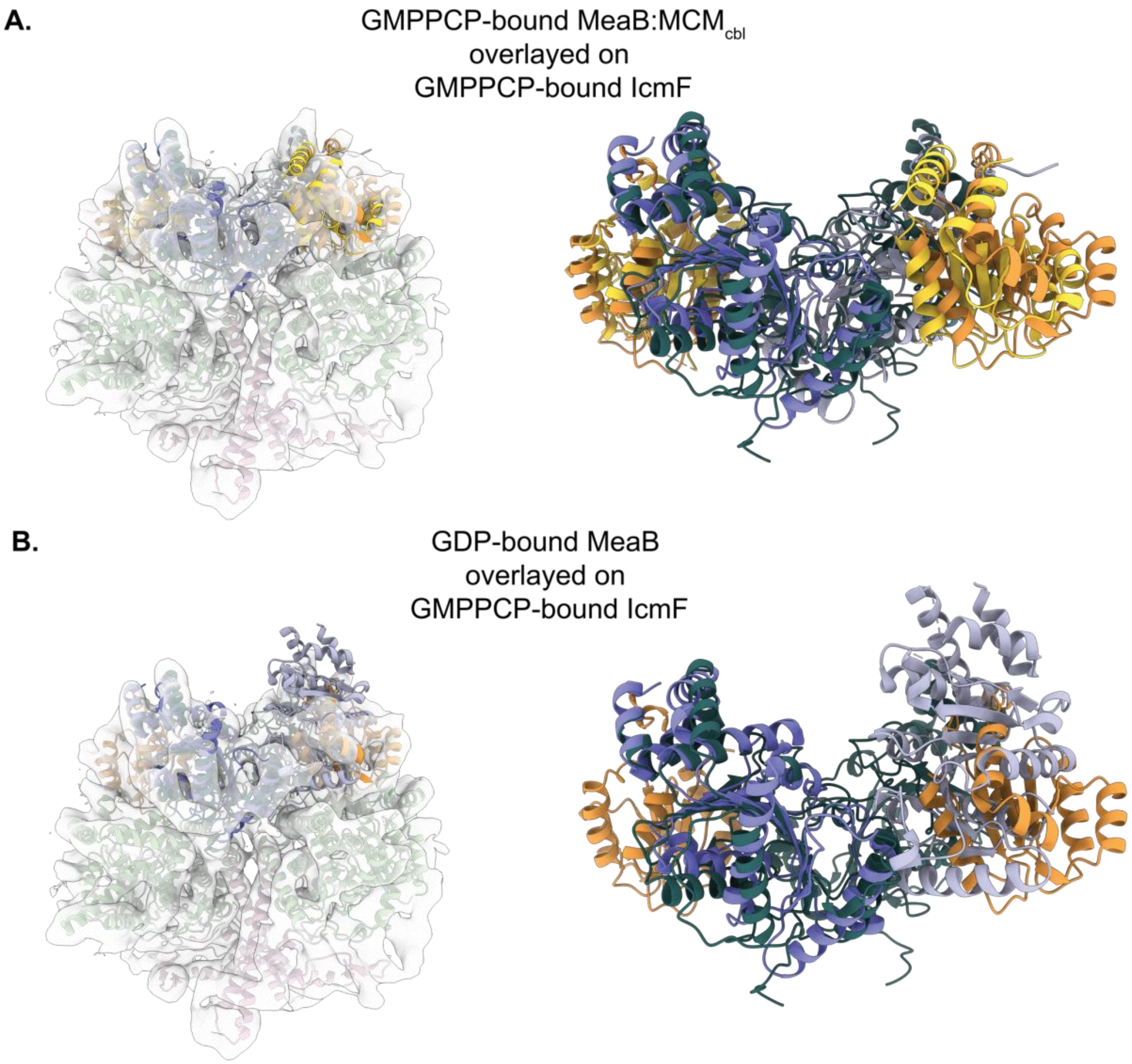

Figure S6. The active conformation of MeaB has good agreement with IcmF cryo-EM data. **A.** Superimposition of MeaB:MCM_{Cbl} complex (MeaB in purple/light purple; MCM_{Cbl} in yellow/orange) (PDB 8DPB) (33) aligned to wt IcmF + GMPPCP structure (colored as in **Fig. 7B**) with cryo-EM map (left) and without map (right). Dimerization domain of MeaB is not shown for clarity in either panel, and IcmF substrate-binding domains and linker regions are not shown on right for clarity. **B.** Superimposition of GDP-bound MeaB (purple/light purple) (PDB 2QM7) (32) aligned to the wt IcmF + GMPPCP structure with cryo-EM map (left) and without map (right). Dimerization domain of MeaB is not shown for clarity in either panel, and IcmF substrate-binding domains and linker regions are not shown on right for clarity.

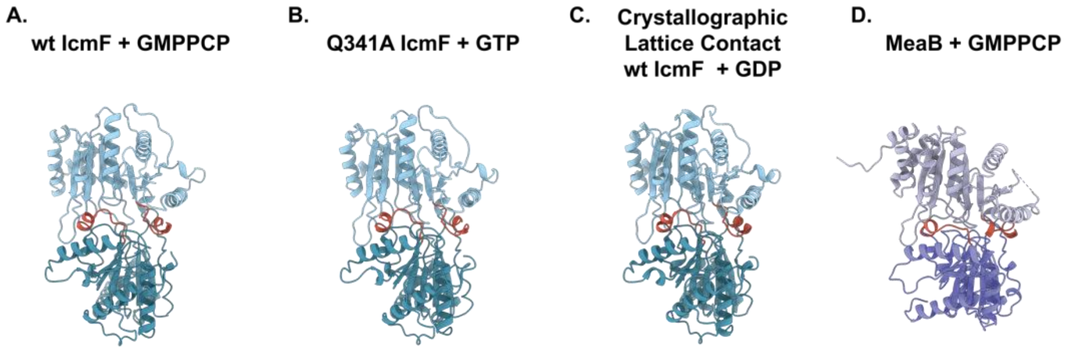

Figure S7. Comparisons of the G-protein domain interface from the IcmF cryo-EM structures to previous structures. **A.** The arrangement of the G-protein domains from the wt IcmF + GMPPCP cryo-EM reconstruction. The switch III residues are colored red-orange and are present at the interface. **B.** The arrangement of the G-protein domains from the Q341A IcmF + GTP cryo-EM reconstruction. The switch III residues are colored red-orange and are present at the interface. **C.** The arrangement of the G-protein domains of wt IcmF from the crystallographic lattice contact (PDB 4XC6) (29) of wt IcmF in the presence of GDP. The switch III residues are colored red-orange and are present at the interface. **D.** The arrangement of the G-protein dimer of MeaB from *M. extorquens* in the presence of the Cbl-binding domain of its target mutase and GMPPCP (PDB 8DPB) (33). The corresponding switch III residues are colored orange and are present at the interface. The C-terminal dimerization helices are not shown for clarity.

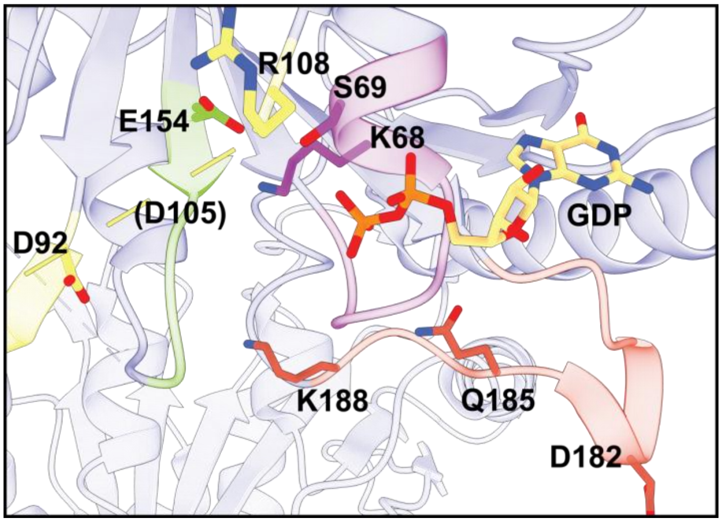

Figure S8. The nucleotide binding site of MeaB bound to GDP. The G-protein chaperone MeaB (PDB 2QM7) (32) (purple ribbons) has conserved motifs indicative of its class of P-loop GTPases as labeled and an additional switch III motif (red orange) implicated in GTP hydrolysis which is necessary for its maturation role. The nucleotide binding site with GDP bound. Residues from the phosphate binding loop (purple sticks: K68 and S69), switch I region (yellow sticks: D92, R108), switch II region (green sticks: E154) and the switch III region (red-orange sticks: K188, Q185, D182) shown to highlight the different interactions of the GDP in comparison to GMPPCP and Mg^{2+} bound as shown in the inset of **Fig. 1C**.

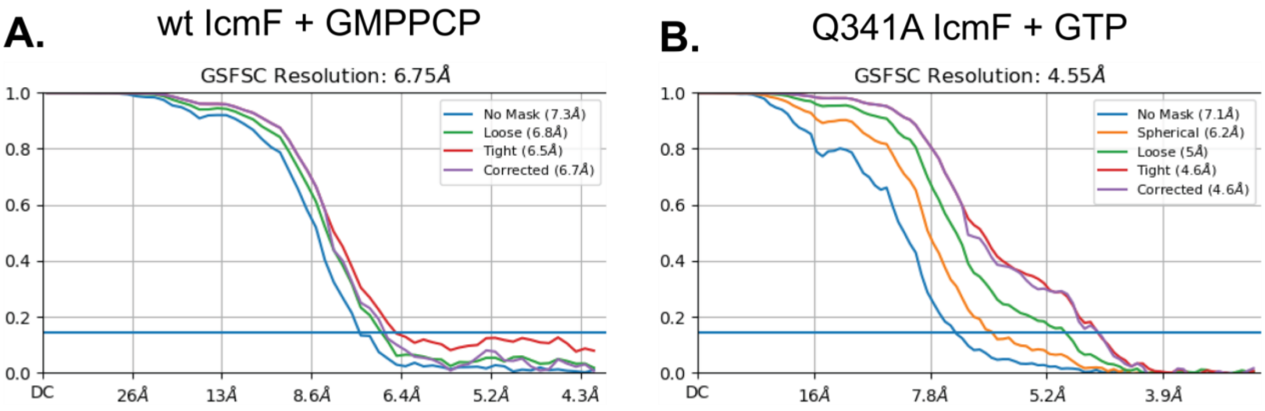

Figure S9. Fourier shell correlation analysis of the two cryo EM reconstructions. **A.** Fourier shell correlation (FSC) plots for the wt IcmF + GMPPCP model indicating the FSC as a function resolution with no mask (blue), a loose mask (green), a tight mask (red) and the corrected mask (purple). **B.** Q341A IcmF + GTP model indicating the Fourier shell correlation as a function of resolution with no mask (blue), a spherical mask (orange), a loose mask (green), a tight mask (red) and the corrected mask (purple). The masks used in the refinements and in the plots were generated by CryoSPARC.

Table S1. Imaging parameters and 3D reconstruction

**Dataset**	**wt IcmF + GMPPCP**	**Q341A IcmF + GTP**
Microscope	FEI Talos Arctica G2	FEI Talos Arctica G2
Camera	Falcon 3EC	Falcon 3EC
Acceleration voltage (kV)	200	200
Magnification (×)	73,000	92,000
Pixel size (Å)	2.0143	1.5998
Defocus range (μm)	1.2 - 3.1	1.2 - 3.1
Number of frames	14	14
Exposure time (s)	3.99	3.99
Total exposure (e^-/Å)	41.81	58.71
Total micrographs collected	602	673
Automation software	EPU	EPU
Particles	138,936	59,335
Symmetry imposed	n/a	n/a
Unmasked resolution at 0.5/0.143 FSC (Å)	8.5/7.3	8.2/7.1
Masked resolution at 0.5/0.143 FSC (Å)	8.1/6.7	6.3/4.6
